# Kif17 phosphorylation regulates photoreceptor outer segment turnover

**DOI:** 10.1186/s12860-018-0177-9

**Published:** 2018-11-20

**Authors:** Tylor R. Lewis, Sean R. Kundinger, Brian A. Link, Christine Insinna, Joseph C. Besharse

**Affiliations:** 1 Department of Cell Biology, Neurobiology, and Anatomy Medical College of Wisconsin, Milwaukee, WI USA; 2 Department of Ophthalmology and Visual Sciences Medical College of Wisconsin, Milwaukee, WI USA; 3 Laboratory of Cell and Developmental Signaling National Cancer Institute-Frederick, Frederick, MD USA

**Keywords:** Retina, Photoreceptor, Intraflagellar transport, Kinesin, Disc shedding, Phagocytosis, Cilia

## Abstract

**Background:**

KIF17, a kinesin-2 motor that functions in intraflagellar transport, can regulate the onset of photoreceptor outer segment development. However, the function of KIF17 in a mature photoreceptor remains unclear. Additionally, the ciliary localization of KIF17 is regulated by a C-terminal consensus sequence (KRKK) that is immediately adjacent to a conserved residue (mouse S1029/zebrafish S815) previously shown to be phosphorylated by CaMKII. Yet, whether this phosphorylation can regulate the localization, and thus function, of KIF17 in ciliary photoreceptors remains unknown.

**Results:**

Using transgenic expression in zebrafish photoreceptors, we show that phospho-mimetic KIF17 has enhanced localization along the cone outer segment. Importantly, expression of phospho-mimetic KIF17 is associated with greatly enhanced turnover of the photoreceptor outer segment through disc shedding in a cell-autonomous manner, while genetic mutants of *kif17* in zebrafish and mice have diminished disc shedding. Lastly, cone expression of constitutively active tCaMKII leads to a *kif17*-dependent increase in disc shedding.

**Conclusions:**

Taken together, our data support a model in which phosphorylation of KIF17 promotes its photoreceptor outer segment localization and disc shedding, a process essential for photoreceptor maintenance and homeostasis. While disc shedding has been predominantly studied in the context of the mechanisms underlying phagocytosis of outer segments by the retinal pigment epithelium, this work implicates photoreceptor-derived signaling in the underlying mechanisms of disc shedding.

**Electronic supplementary material:**

The online version of this article (10.1186/s12860-018-0177-9) contains supplementary material, which is available to authorized users.

## Background

Cilia, organelles that project from the cell body and contain a microtubule-based axoneme, have numerous roles in development, signal transduction, and cell motility [1] and have garnered widespread interest due to associations with a widespread set of disorders termed ciliopathies [2]. Of particular interest, photoreceptors are widely studied based on their modified primary cilium called the outer segment (OS) that contains the components required for phototransduction [3]. The kinesin-2 family member, KIF17, is expressed in photoreceptors [4] and may regulate the onset of OS morphogenesis [5]. However, there has been no evidence of a role for KIF17 in a mature photoreceptor [6]. As KIF17 can accumulate at the ciliary tip of photoreceptors [7], like mammalian cell lines [6, 8–10], it is possible that KIF17 may have some role in distal tip maintenance. Interestingly, the distal tip of the OS is phagocytized by the opposing retinal pigment epithelium (RPE) daily in a process called disc shedding [11]. The molecular mechanisms underlying disc shedding have been predominantly studied at the level of the RPE, but exposed phosphatidylserine (PS) on the tips of OS precedes disc shedding [12], suggesting photoreceptor signaling contributes to the underlying mechanism of disc shedding.

**Fig. 1:**
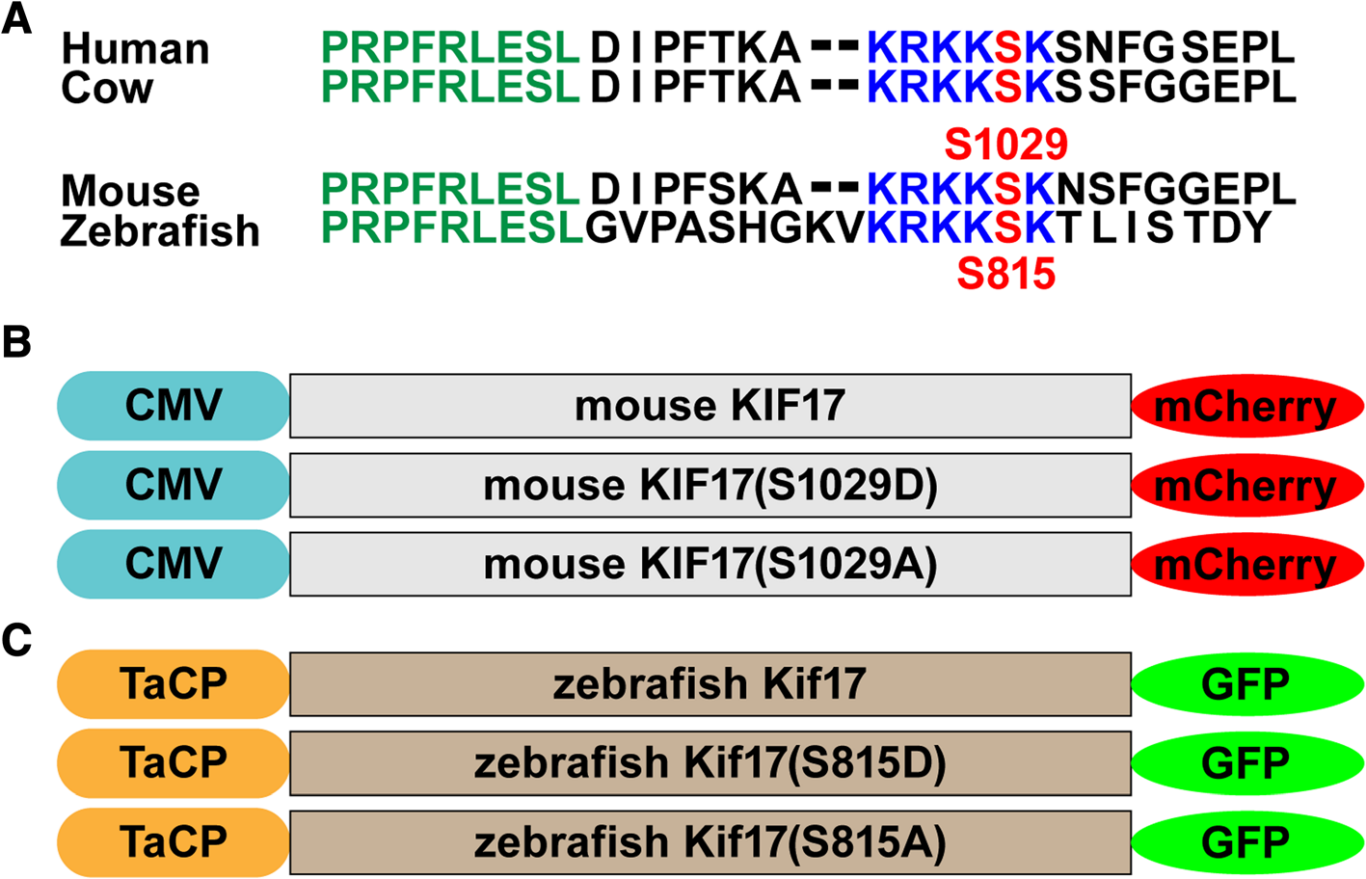
Schematic of transgenic phospho-mutant KIF17 constructs. **a** The amino acid sequence of the C-terminus of KIF17 in various species is depicted. Homologous sequences are shown in color (green, blue, and red). The green conserved sequence has been shown to interact with the IFT-B complex [9], while the blue conserved NLS can regulate ciliary localization [8]. The conserved serine (red), mouse S1029/zebrafish S815, has been shown to be phosphorylated by CaMKII [16]. **b** For mammalian cell culture experiments (Additional file 1: Figures S1, S2), three different mCherry-tagged constructs of mouse KIF17 under control of the ubiquitous CMV promoter were generated: wild-type KIF17, phospho-mimetic KIF17(S1029D), and phospho-deficient KIF17(S1029A). **c** For zebrafish cone photoreceptor experiments, three different GFP-tagged constructs of zebrafish under control of the cone-specific transducin-α promoter (TaCP) were generated: wild-type Kif17, phospho-mimetic Kif17(S815D), and phospho-deficient Kif17(S815A)

Although the ciliary membrane is continuous with the plasma membrane, the transition zone at the base of the cilium is believed to function as a ciliary gate involved in regulating protein entry and exit [13]. While there has been conflicting evidence regarding the permeability barrier at the base of cilia [14, 15], KIF17 has a putative nuclear localization signal (NLS) (Fig. 1a) that can regulate its ciliary entry through a classical nuclear import mechanism [8, 9]. Further, calcium/calmodulin-dependent protein kinase II, or CaMKII, has been shown to phosphorylate the C-terminus of KIF17 to regulate binding and release of Mint1, a scaffolding protein for motor cargo, in neurons [16]. Interestingly, this conserved phosphorylation site lies immediately adjacent to the NLS in KIF17 (Fig. 1a). In this work, we were interested in identifying the function of this conserved phosphorylation site on Kif17 localization and function in photoreceptors. Given the proximity to the NLS, we originally hypothesized that phosphorylation regulates the ciliary entry of Kif17. Further, given the accumulation of Kif17 at the ciliary tip of photoreceptors [7], we hypothesized that Kif17 phosphorylation could regulate a possible role of Kif17 in disc shedding.

Functional roles of phosphorylation are often studied through specific mutagenesis of phosphorylation sites, which can be mutated to aspartic acid (D) or glutamic acid (E) to chemically imitate a constitutively phosphorylated residue or alanine (A) to imitate a constitutively un-phosphorylated residue [17–20]. While not a direct assessment of the function of phosphorylation of endogenous protein, phospho-mutants have been previously used to study KIF17 function and localization [16, 21]. In this work, we express three different forms of GFP-tagged zebrafish Kif17 (wild-type, phospho-mimetic S815D, and phospho-deficient S815A) (Fig. 1c) in cone photoreceptors and show that the accumulation of Kif17 in the zebrafish OS is controlled through this phosphorylation site. Cone expression of phospho-mimetic Kif17 leads to a three-fold increase in disc shedding, while the genetic mutant *kif17*^*mw405*^ has diminished disc shedding. Additionally, cone expression of a constitutively active CaMKII leads to a two-fold increase in disc shedding in wild-type, but not *kif17*^*mw405*^ mutant fish. Taken together, this work supports a model in which phosphorylation of Kif17 regulates its accumulation in cilia. In cone photoreceptors, phosphorylated Kif17 in the OS participates in a cell-autonomous process to promote disc shedding.

## Results

### Phospho-mimetic mouse KIF17 has enhanced ciliary localization

Published data suggest that the conserved NLS in the C-terminus of KIF17 can regulate the ciliary localization of KIF17 through a classical nuclear import mechanism [8]. Although KIF17 has been predominantly studied as a ciliary or dendritic motor [22], it has been reported to localize to the nucleus and function in transcriptional regulation [23, 24]. Yet the context under which a single conserved NLS can function to localize a protein to either the nucleus or cilium remains unclear. Interestingly, a conserved phosphorylation site (S1029 in mice and S815 in zebrafish) lies immediately adjacent to this NLS (Fig. 1a) and has been shown to be phosphorylated by CaMKII in neurons [16]. Before investigating the function of this phosphorylation site in photoreceptors, we first sought to investigate this phosphorylation site with regards to ciliary and nuclear localization in ciliated mammalian cells and generated three different constructs of mouse KIF17 for mammalian cell expression with the CMV promoter: KIF17-mCherry, phospho-mimetic KIF17(S1029D)-mCherry, and phospho-deficient KIF17(S1029A)-mCherry (Fig. 1b). We expressed these constructs in four different ciliated mammalian cell lines: LLC-PK1, a pig kidney epithelial line; HEK-293, a human kidney line; hTERT-RPE1, a human retinal pigment epithelial line; and IMCD3, a mouse kidney line. With transient transfection of serum-starved cells, we can determine the extent of ciliary localization of each transgene with co-labeling of acetylated α-tubulin, which labels the axoneme, or nuclear localization with co-labeling of Hoechst (Additional file 1: Figure S1A). The frequency of ciliary localization of the phospho-mimetic KIF17(S1029D)-mCherry was significantly increased between two- to four-fold over the phospho-deficient KIF17(S1029A)-mCherry in all cell lines studied (Additional file 1: Figure S1B). Interestingly, the ciliary localization of wild-type KIF17-mCherry was never significantly different from the phospho-deficient KIF17(S1029A)-mCherry with both having a baseline level of ciliary localization, suggesting that phosphorylation enhances ciliary localization but does not absolutely control ciliary entry.

To analyze nuclear localization, we calculated the ratio of average mCherry signal intensity between the nucleus and cytoplasm (Additional file 1: Figure S2). In two of the four cell lines (LLC-PK1 and HEK-293), there were no effects on transgene localization between the nucleus and cytoplasm despite there being a ciliary localization difference. However, in hTERT-RPE1 and IMCD3 cells, the phospho-mimetic KIF17(S1029D)-mCherry had a decreased nuclear to cytoplasmic ratio, suggesting inhibition of nuclear localization as compared to either wild-type KIF17-mCherry or phospho-deficient KIF17(S1029A)-mCherry. Yet, only in hTERT-RPE1 cells did the nuclear to cytoplasmic ratio exceed one. Taken together, these data support a model in which phosphorylation of the conserved S1029 residue of mouse KIF17 strongly promotes ciliary localization in four different cell lines. In contrast, nuclear localization of KIF17 may be negatively regulated by phosphorylation depending on cell type.

### Phospho-mimetic zebrafish Kif17 has enhanced OS localization

**Fig. 2:**
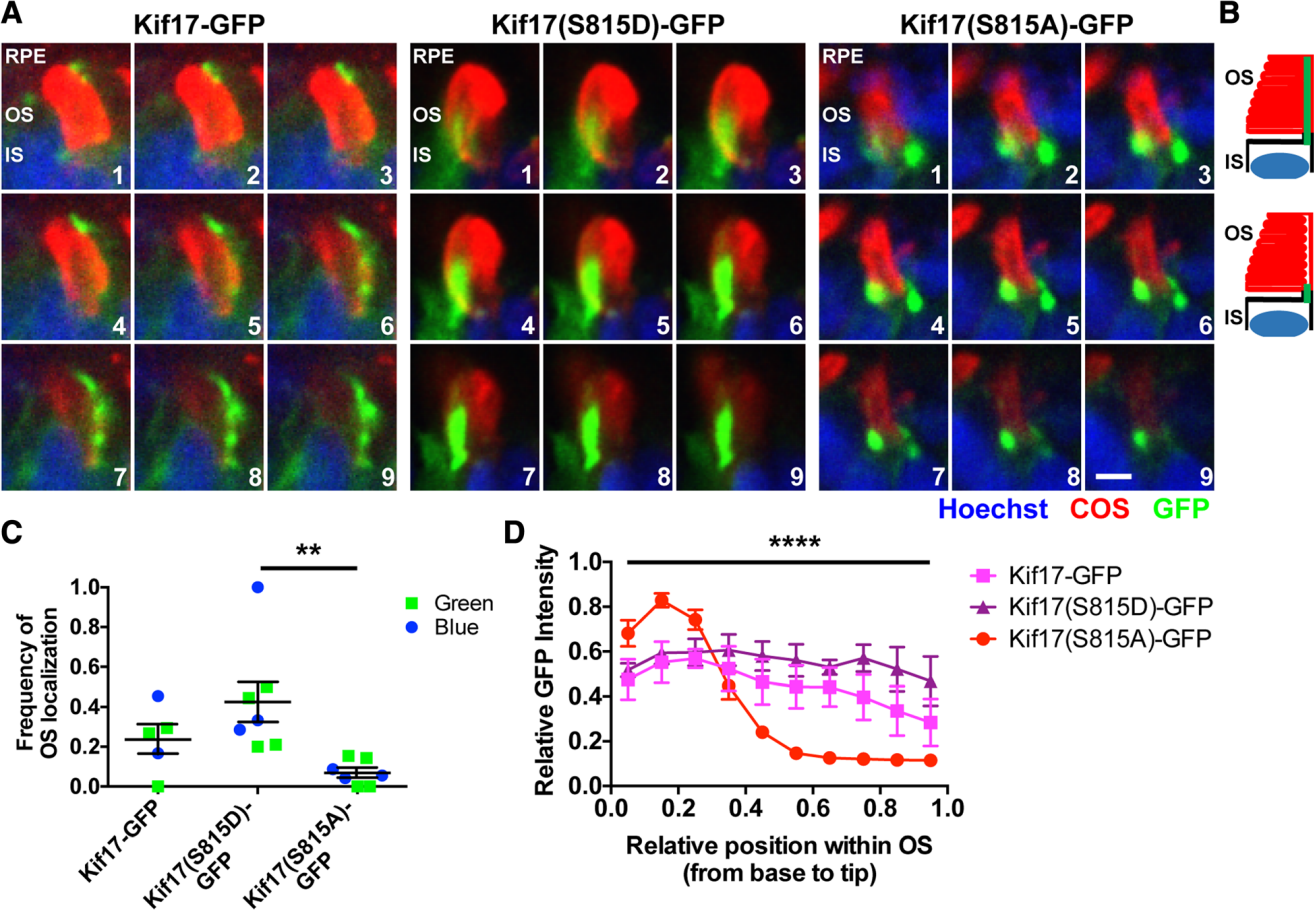
Phospho-mutations of S815 regulate photoreceptor OS localization of Kif17. **a** 5 dpf larvae previously injected at the one cell stage with one of three different transgenic constructs under control of the TaCP promoter for expression in cone photoreceptors: Kif17-GFP (left), phospho-mimetic Kif17(S815D)-GFP (middle), and phospho-deficient Kif17(S815A)-GFP (right) were stained with Hoechst (blue) to label nuclei and blue cone opsin (red) to label cone OS (COS). A z-series of confocal images was taken with 0.2 μm steps through the depth of the photoreceptor OS. Shown are montages of sequential images in the z-series revealing that Kif17(S815D)-GFP exhibits significant accumulation in the OS while Kif17(S815A)-GFP accumulates at the base of the OS. Note that in cone photoreceptors, the ciliary axoneme extends along the side of the opsin-containing discs. Scale bar is 1 μm. **b** Models depicting structure of the photoreceptor OS imaged in Fig. 2a. There appears to be two subsets of GFP localization: GFP localizing along the length of the OS (top) and GFP accumulating at the base of the OS (bottom). **c** Quantification of the frequency of OS accumulation of Kif17-GFP (*n* = 5 total injected larvae: 3 stained for green opsin, 2 stained for blue opsin; 60 cells), Kif17(S815D)-GFP (*n* = 7: 4 green, 3 blue; 62 cells), and Kif17(S815A)-GFP (*n* = 7: 4 green, 3 blue; 126 cells) when co-labeled with either green opsin (square) or blue opsin (circle). **d** Line intensity analysis of the GFP signal for Kif17-GFP (pink line, *n* = 5 total injected larvae: 3 stained for green opsin, 2 stained for blue opsin; 17 cells), Kif17(S815D)-GFP (purple line, *n* = 5: 3 green, 2 blue; 17 cells), and Kif17(S815A)-GFP (red line, *n* = 5: 3 green, 2 blue; 25 cells). Two-way ANOVA was performed to determine significance in the different interaction between GFP signal intensity pattern and transgene (*p* < 0.0001, ****)

While phosphorylation of KIF17 may regulate ciliary localization in mammalian cells, our main interest was in investigating the effect of KIF17 phosphorylation in ciliary photoreceptor OS in vivo. We generated comparable zebrafish Kif17 constructs specifically for cone photoreceptor expression with the cone-specific transducin-α (*gnat2*) promoter, also known as TaCP: Kif17-GFP, phospho-mimetic Kif17(S815D)-GFP, and phospho-deficient Kif17(S815A)-GFP **(**Fig. 1c**)**. While previous work shows that Kif17-GFP can accumulate at the distal tip of the OS [7], we used transient injections of each of these constructs into wild-type zebrafish embryos to quantify localization within the OS of two sub-types of zebrafish cones: blue and green absorbing cones. Comparable to the mammalian cell work, phospho-mimetic Kif17(S815D)-GFP accumulated throughout the OS, while phospho-deficient Kif17(S815A)-GFP appeared to accumulate at the base of the OS (Fig. 2a, Additional file 1: Figure S3). The accumulation of phospho-mimetic Kif17(S815D)-GFP in the OS was seven-fold greater than that of phospho-deficient Kif17(S815A)-GFP (Fig. 2c). Additionally, line intensity analysis reveals that phopsho-mimetic Kif17(S815D)-GFP localizes evenly along the entire length of the OS, while phospho-deficient Kif17(S815A)-GFP exhibited a specific accumulation at the base of the OS (Fig. 2d). We did not observe any evidence of nuclear localization of the zebrafish Kif17-GFP constructs (Additional file 1: Figure S3). These data further support a model in which phosphorylation of Kif17 leads to an enhanced localization in the ciliary photoreceptor OS.

### KIF17 promotes cell-autonomous disc shedding

While we have previously shown a developmental role of KIF17 in regulating the onset of OS morphogenesis [5], there is no evidence of a role of KIF17 in mature photoreceptors [6]. We hypothesized that investigating the temporal expression patterns of *Kif17* in mature photoreceptors throughout the diurnal light:dark cycle could shed light on a potential function of KIF17. We performed qPCR on mouse retinas that were collected every four hours during a 24 h period beginning at light onset, indicated as Zeitgeber Time (ZT) 0, through dark onset (ZT 12), and stopping at ZT 20, or 20 h following light onset. Interestingly, there is a single peak in *Kif17* expression in the mouse retina at ZT 4, just following light onset, which subsequently returns to a basal level (Additional file 1: Figure S4A). We also performed qPCR on 14 dpf zebrafish eyes that were collected at nine discrete timepoints over a 24 h period beginning at light onset. In contrast to the mice, which are kept on a 12 h:12 h light:dark cycle, the zebrafish are maintained on a 14 h:10 h light:dark cycle, so that ZT 14 indicates dark onset. While there is an increase in zebrafish *kif17* expression immediately after light onset (ZT 1.5), there is a second, more intense peak just following dark onset (ZT 16 and ZT 18) (Additional file 1: Figure S4B). While *Kif17* appears to be rhythmically expressed in both mouse and zebrafish, mice have a single peak of expression associated with light onset, whereas zebrafish have two peaks of expression associated with both light and dark onset.

Due to the association of *kif17* expression with both light and dark onset in zebrafish, we hypothesized that Kif17 could be involved in disc shedding, the daily process of OS turnover in which RPE cells adjacent to the OS phagocytize and degrade shed OS tips. To analyze disc shedding, we performed transmission electron microscopy (TEM) on 5dpf zebrafish larvae that had been injected with the cone-expressing plasmids encoding either one of the three Kif17 transgenes or a soluble GFP control. Lacking transposase, the plasmids do not incorporate into the genome but rather facilitate episomal expression of transgenes. Interestingly, transient expression of the phospho-mimetic Kif17(S815D)-GFP was sufficient to drive an increase in the number of phagosomes compared to wild-type Kif17, phospho-deficient Kif17, or the soluble GFP control (Additional file 1: Figure S5).

**Fig. 3:**
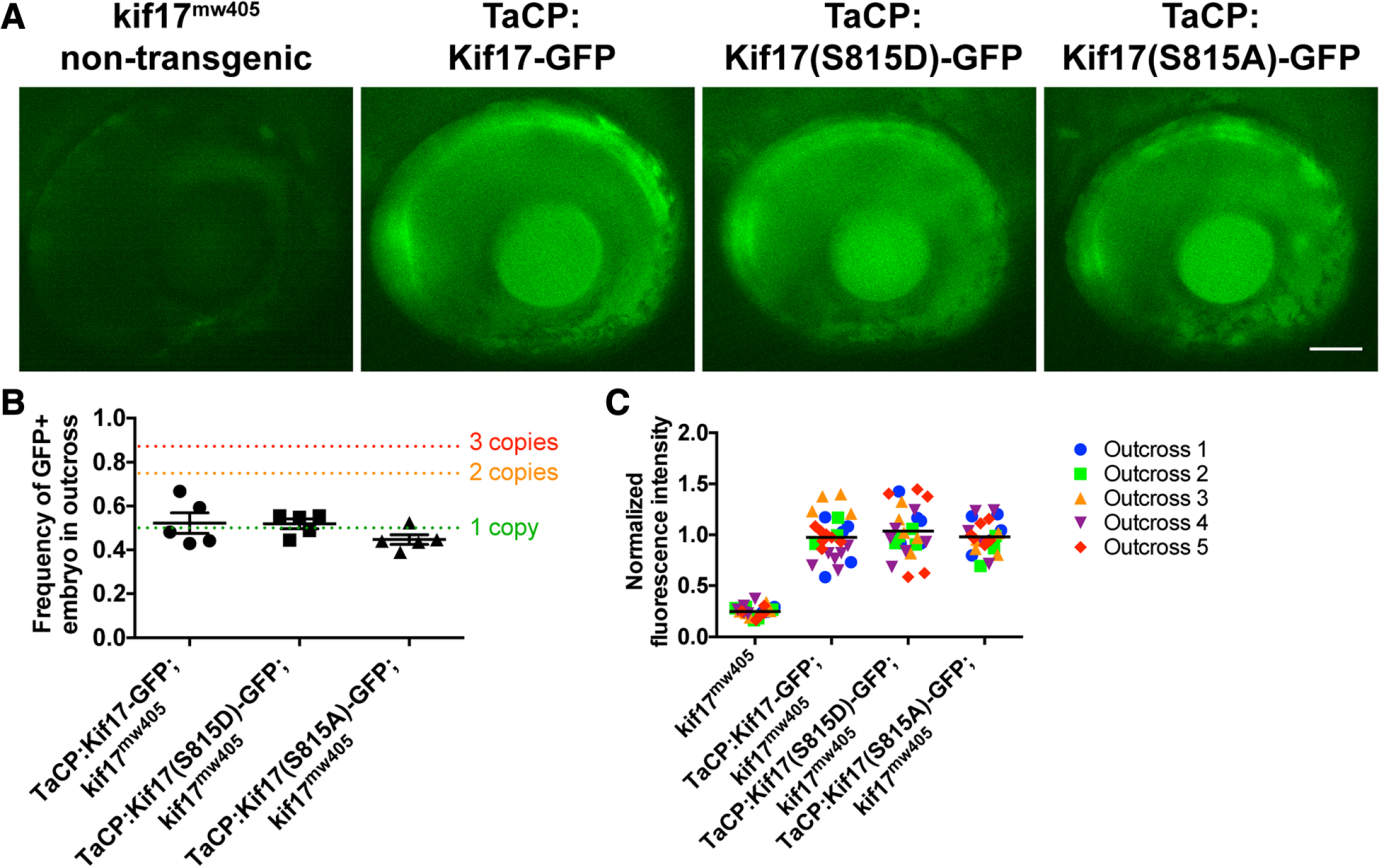
Establishment of stable lines of Kif17 transgenic zebrafish. **a** The three stable transgenic lines of zebrafish were outcrossed to *kif17*^*mw405*^ zebrafish. At 4dpf, zebrafish embryos were screened with epifluorescence to ensure virtually identical levels of expression among the three lines: Kif17-GFP, Kif17(S815D)-GFP, and Kif17(S815A)-GFP. Of note, the bright band of fluorescence at the back of the eye corresponds to the cone photoreceptors that are specifically expressing each of the three transgenes through TaCP, the cone-specific promoter. Additionally, each of the transgenic zebrafish have a significantly higher level of fluorescence than the autofluorescence observed in non-transgenic *kif17*^*mw405*^ zebrafish. For any experiment using the stable transgenic lines of zebrafish, screening is performed to verify the virtually identical level of expression. Scale bar is 50 μm. **b** For generation of the three stable transgenic lines of zebrafish, several generations of outcrossing to *kif17*^*mw405*^ zebrafish were performed to ensure there was only a single genomic insert of each transgene. Following independent assortment and Mendelian inheritance, the frequency of transgene inheritance in an outcross can be used to determine the insert or copy number of each transgene according to: $Frequency=1-\frac{1}{2^2}$, where *n* = copy number. Thus, a single insert would be inherited 50% of the time (green dotted line), two inserts would be 75% of the time (orange dotted line), and three inserts would be 87.5% of the time (red dotted line). Of note, the frequency of transgene inheritance for each of the three stable lines, TaCP:Kif17-GFP (*n* = 5 outcrosses, 203 total embryos screened), TaCP:Kif17(S815D)-GFP (*n* = 5 outcrosses, 231 embryos), and TaCP:Kif17(S815A)-GFP (*n* = 5 outcrosses, 279 embryos), is ~ 50%, suggesting that each of the stable transgenic lines contain only a single insert. One-way ANOVA was performed to show that there is no statistical significant difference among inheritance of each transgene (*p* = 0.2163). One sample t-test was performed to show that frequencies of TaCP:Kif17-GFP (*p* = 0.6579), TaCP:Kif17(S815D)-GFP (*p* = 0.4510), and TaCP:Kif17(S815A)-GFP (*p* = 0.0706) inheritance were not significantly different from 50%. **c** In addition to ensuring each of the stable lines was a single insert, we selected for stable transgenic lines that had a virtually identical level of fluorescence among them. The fluorescence intensity of each of the transgenes as well as a non-transgenic *kif17*^*mw405*^ zebrafish (*n* = 5 outcrosses, 25 total embryos each) was measured and normalized to the average fluorescence intensity of TaCP:Kif17-GFP. One-way ANOVA was performed to show that there is a statistical significant difference in fluorescence levels among the transgenic fish and the non-transgenic fish (*p* < 0.0001). A post-hoc Bonferroni analysis to compare groups was performed to show no significant differences between any of the transgenic fish (*p* > 0.9999), but a significant increase in fluorescence of the transgenic fish compared to the autofluorescence of a non-transgenic fish (*p* < 0.0001, ****)

Because of the potential for interactions of the Kif17 transgenes with endogenous wild-type Kif17 and the transient and mosaic nature of expression of injected plasmids, we generated stable lines of each of our cone-expressing transgenes (Kif17-GFP, phospho-mimetic Kif17(S815D)-GFP, and phospho-deficient Kif17(S815A)-GFP) on a mutant background called *kif17*^*mw405*^ lacking endogenous Kif17 [5]. In the *Tol2* transposon system frequently used in zebrafish transgenesis, the injected transgene is integrated into random, oftentimes multiple locations of the genome, requiring several generations of out-crossing to generate transgenic lines that exhibit stable transgene expression that is inherited in a Mendelian fashion [25, 26]. This is particularly necessary in the case of comparing phenotypes of transgenic expression between different lines, such as for the phopsho-mutant Kif17-GFP lines. Following several generations of out-crossing, we validated that each of the three transgenic lines with cone expression of Kif17-GFP, phospho-mimetic Kif17(S815D)-GFP, or phospho-deficient Kif17(S815A)-GFP contained only a single insert through analyzing inheritance frequency of an outcross (Fig. 3a,b). As expected, only ~ 50% of offspring of each of the transgenic lines exhibited positive fluorescence, consistent with Mendelian inheritance of a single insert. Next, we analyzed the fluorescence intensity of each of the three transgenes in 4dpf embryos from each transgenic line and show that they are comparable across each of the three transgenic lines (Fig. 3a,c), suggesting that the expression of each transgene is equal.

**Fig. 4:**
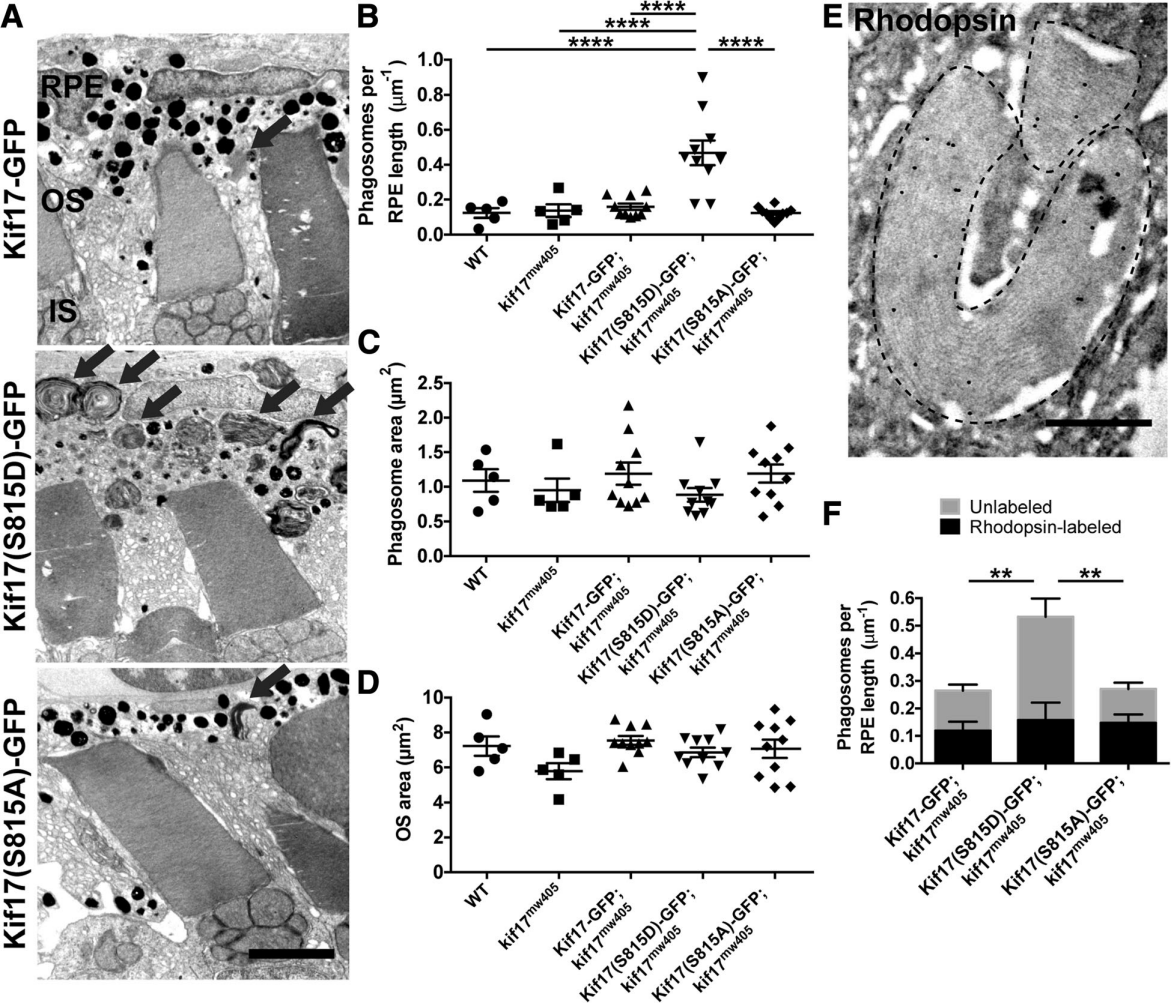
Phospho-mimetic Kif17(S815D) increases disc shedding. **a** TEM images of 7dpf *kif17*^*mw405*^ larvae that are stably expressing either Kif17-GFP (upper), phospho-mimetic Kif17(S815D)-GFP (middle), or phospho-deficient Kif17(S815A)-GFP (lower) in cones collected at ZT 1.5. Black arrows indicate phagosomes. Scale bar is 2 μm. IS is inner segment. **b** Quantification of the number of phagosomes for wild-type (*n* = 5 larvae, 1273 μm of RPE length); *kif17*^*mw405*^ (*n* = 5, 1170 μm of RPE length); Kif17-GFP, *kif17*^*mw405*^ (*n* = 10, 2677 μm of RPE length); Kif17(S815D)-GFP, *kif17*^*mw405*^ (*n* = 10, 2938 μm of RPE length); and Kif17(S815A)-GFP, *kif17*^*mw405*^ (*n* = 10, 2875 μm of RPE length) larvae. **c** Quantification of the area of phagosomes for wild-type (*n* = 5 larvae, 403 phagosomes); *kif17*^*mw405*^ (*n* = 5, 392 phagosomes); Kif17-GFP, *kif17*^*mw405*^ (*n* = 10, 530 phagosomes); Kif17(S815D)-GFP, *kif17*^*mw405*^ (*n* = 10, 1601 phagosomes); and Kif17(S815A)-GFP, *kif17*^*mw405*^ (*n* = 10, 547 phagosomes) larvae. **d** Quantification of OS size for wild-type (*n* = 5 larvae, 268 OS); *kif17*^*mw405*^ (n = 5, 255 OS); Kif17-GFP, *kif17*^*mw405*^ (*n* = 10, 615 OS); Kif17(S815D)-GFP, *kif17*^*mw405*^ (*n* = 10, 566 OS); and Kif17(S815A)-GFP, *kif17*^*mw405*^ (*n* = 10, 605 OS) larvae. **e** Immunogold labeling of a 7 dpf TaCP:Kif17-GFP zebrafish retina with K62-171c, an antibody against bovine rhodopsin showing a positive labeling of phagosomes (outlined with dashed lines), as also depicted in Additional file 1: Figure S7. Scale bar is 0.5 μm. **f** Analysis of immunogold labeling of rhodopsin-containing or unlabeled phagosomes in 7 dpf Kif17-GFP, *kif17*^*mw405*^ (*n* = 5 larvae, 1031 μm of RPE); Kif17(S815D)-GFP, *kif17*^*mw405*^ (*n* = 5, 1268 μm of RPE); and Kif17(S815A)-GFP, *kif17*^*mw405*^ (*n* = 5, 889 μm of RPE) larvae. Two-way ANOVA was performed to determine significance in the interaction between the transgene expressed and the concentration of rod or cone phagosomes (*p* = 0.0264, *) as well as significance in the total number of phagosomes between transgenes (*p* = 0.0071, **). Bonferroni’s multiple comparisons post-hoc test was performed to determine significance in the differences of unlabeled phagosomes between Kif17-GFP and Kif17(S815D)-GFP (*p* = 0.0033, **) and between Kif17(S815A)-GFP and Kif17(S815D)-GFP (*p* = 0.0013, **). There were no differences in numbers of rhodopsin-labeled phagosomes among the transgenes

To measure disc shedding in the three transgenic lines, we collected 7dpf larvae at ZT 1.5 and performed TEM to analyze phagosomes (Fig. 4a). There was a dramatic three-fold increase in the number of phagosomes observed in the RPE in retinas with cone expression of Kif17(S815D)-GFP as compared to either Kif17-GFP or Kif17(S815A)-GFP (Fig. 4b), while the average size of phagosomes was unchanged (Fig. 4c). Surprisingly, while there was a significant increase in disc shedding associated with Kif17(S815D)-GFP expression in cones, there was no decrease in average OS area (Fig. 4d), suggesting that the excess in disc shedding may be compensated by increased OS morphogenesis. However, an upregulation of opsin mRNA expression, which encodes the major protein of the OS, was not observed (Additional file 1: Figure S6).

Because our TEM analysis does not distinguish between phagosomes from either cone or rod photoreceptors, we performed immunogold labeling of these transgenic larvae. Using a monoclonal antibody against bovine rhodopsin, K62-171c [27], that also specifically labels zebrafish rhodopsin-containing OS and phagosomes (Fig. 4e, Additional file 1: Figure S7), we show that cone expression of Kif17(S815D)-GFP leads to a three-fold increase in unlabeled phagosomes, presumably from cones, compared to larvae with cone expression of Kif17(S815A)-GFP (Fig. 4f). However, there is no significant change in the number of rhodopsin-labeled phagosomes. Additionally, we specifically labeled cone-derived phagosomes using an antibody against gnat2, the cone-specific transducin-α whose promoter was used to drive expression of the transgenes in this study. Although the immunogold labeling of cone transducin-α labeled both cone OS and phagosomes **(**Additional file 1: Figure S8A), the labeling efficiency was significantly lower than that of the rhodopsin antibody, presumably due to a lower concentration of gnat2 in the OS. Nonetheless, we show that cone expression of Kif17(S815D)-GFP leads to a two-fold increase in cone transducin-α-labeled phagosomes with no significant change in unlabeled phagosomes, presumably from rods (Additional file 1: Figure S8B). Taken together, these data suggest phosphorylation of Kif17 promotes both OS localization and subsequent cone disc shedding in a cell-autonomous manner.

### Loss of KIF17 diminishes disc shedding in zebrafish and mice

**Fig. 5:**
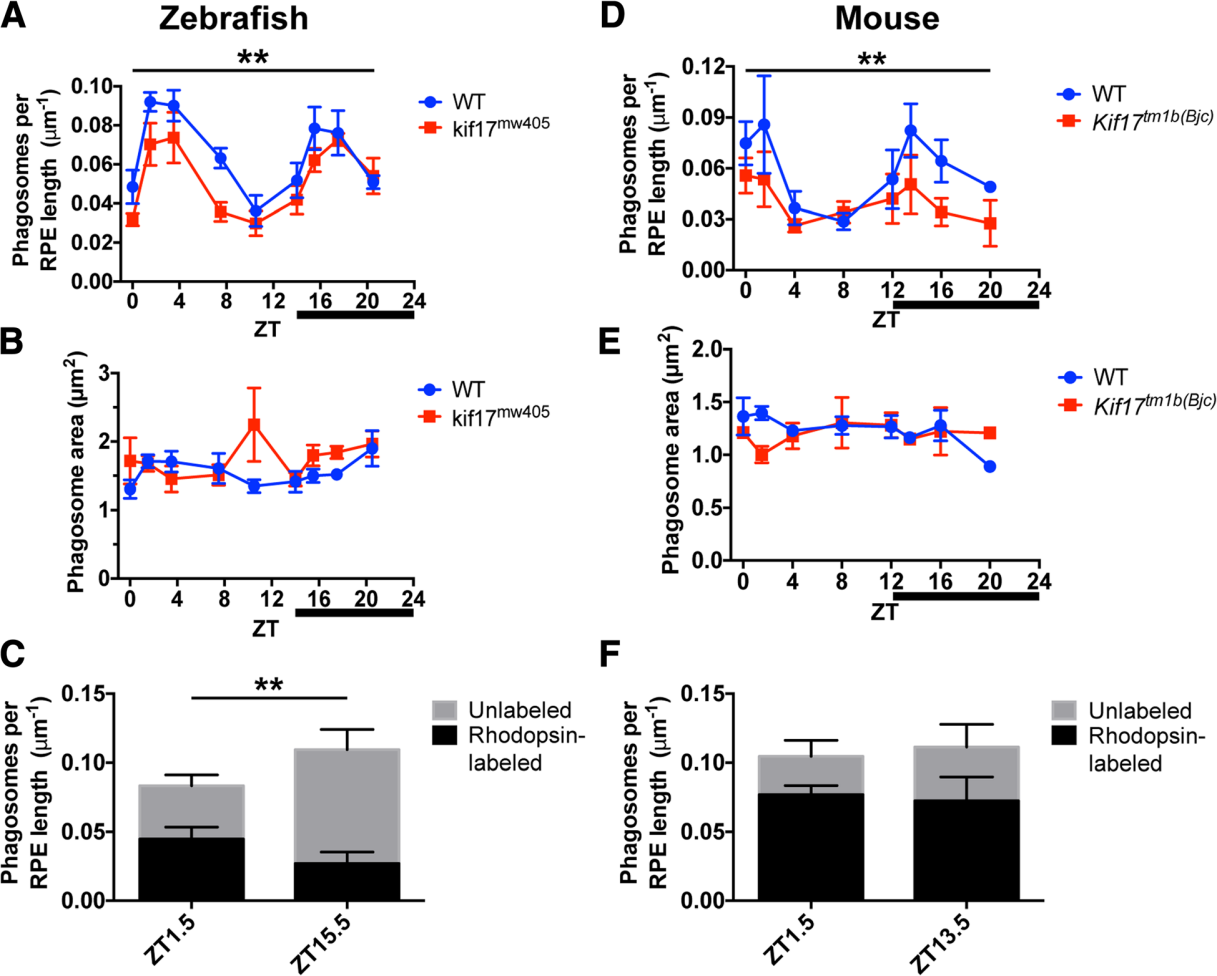
Loss of *kif17* diminishes disc shedding. **a** TEM analysis of phagosome number in 14 dpf wild-type (*n* = 5 larvae at each timepoint, 1400 ± 97 μm of RPE measured at each timepoint) and *kif17*^*mw405*^ (*n* = 5 larvae at each timepoint, 1469 ± 36 μm of RPE measured at each timepoint) zebrafish collected at nine discrete timepoints following light onset (ZT 0). Dark onset begins at ZT 14 (indicated by dark bar). Two-way ANOVA was performed to determine significance in both phagosome number rhythmicity throughout the timepoints (*p* < 0.0001, ****) as well as between genotypes (*p* = 0.0011, **). **b** TEM analysis of phagosome size in 14 dpf wild-type (*n* = 5 larvae at each timepoint, 93 ± 14 phagosomes measured at each timepoint) and *kif17*^*mw405*^ (*n* = 5 larvae at each timepoint, 79 ± 10 phagosomes measured at each timepoint) zebrafish. **c** Immunogold labeling of rhodopsin-containing phagosomes in 14 dpf wild-type retina at either the morning peak of disc shedding, ZT 1.5 (*n* = 5 larvae, 1738 μm of RPE), or the evening peak, ZT 15.5 (*n* = 5, 1341 μm of RPE). Two-way ANOVA was performed to determine significance in the interaction between the time of day and the concentration of rod or cone phagosomes (*p* = 0.0082, **). There were no statistical differences in the total number of phagosomes between the morning or night peak. Bonferroni’s multiple comparisons post-hoc test was performed to determine significance in the increased of unlabeled phagosomes between morning and night (*p* = 0.0158, *). **d** TEM analysis of phagosome number in 2–4 month wild-type (*n* = 3 mice at each timepoint, 979 ± 42 μm of RPE measured at each timepoint) and *Kif17*^*tm1b(Bjc)*^ (*n* = 3 mice at each timepoint, 940 ± 45 μm of RPE measured at each timepoint) mice collected at eight discrete timepoints following light onset (ZT 0). Dark onset begins at ZT 12 (indicated by dark bar). Two-way ANOVA was performed to determine significance in both phagosome number rhythmicity throughout the timepoints (*p* = 0.0300, *) as well as between genotypes (*p* = 0.0096, **). **e** TEM analysis of phagosome size in 2–4 month wild-type (*n* = 3 mice at each timepoint, 54 ± 7 phagosomes measured at each timepoint) and *Kif17*^*tm1b(Bjc)*^ (*n* = 3 mice at each timepoint, 35 ± 4 phagosomes measured at each timepoint) mice. **f** Immunogold labeling of rhodopsin-containing phagosomes with B630 rhodopsin antibody in mouse retina at either the morning peak of disc shedding, ZT 1.5 (*n* = 5 mice, 554 μm of RPE), or the evening peak, ZT 13.5 (*n* = 5, 594 μm of RPE). Two-way ANOVA was performed to determine no significance differences in the interaction between the time of day and the concentration of rod or cone phagosomes (*p* = 0.5700) or in the differences in phagosome number between the morning or night peak (*p* = 0.8076), but there was a significant increase in the total number of rod phagosomes compared to cone phagosomes (*p* = 0.0074, **)

While transgenic cone expression of phospho-mimetic Kif17(S815D)-GFP promotes disc shedding, we sought to further investigate how the loss of *kif17* affects disc shedding. While we did not observe defects in disc shedding in *kif17*^*mw405*^ mutants at the single timepoint (ZT 1.5) analyzed in our experiments with the transgenic fish (Fig. 4b), we proposed that deficiencies of disc shedding in the *kif17*^*mw405*^ zebrafish would be better observed when analyzed throughout the entire day. We collected larvae at 14 dpf, when OS have reached their mature size [5], at the nine timepoints over a 24 h period and performed TEM to image phagosomes in both wild-type and *kif17*^*mw405*^ zebrafish. We find that zebrafish disc shedding is highly rhythmic, with two peaks associated with both light and dark onset. Additionally, there is a significant decrease in the number of phagosomes shed in zebrafish lacking Kif17 (*kif17*^*mw405*^) as compared to controls (Fig. 5a). Although there were changes in the disc shedding rates between genotypes and throughout the day, there was no change in phagosome size (Fig. 5b).

To more carefully analyze the differences in disc shedding patterns between wild-type and *kif17*^*mw405*^ larvae as well as between the morning and evening peaks, we performed spline interpolation, a method of constructing a piecewise polynomial curve to smoothly fit the disc shedding data across all timepoints, for both the wild-type and *kif17*^*mw405*^ phagosome number data (Additional file 1: Figure S9A). For wild-type larvae, the two peaks in the number of phagosomes occurred 2.5 h after light onset and subsequently 2 h after dark onset (Additional file 1: Table S1). In comparison, *kif17*^*mw405*^ mutants had estimated peaks at 2.5 h after light onset, and 3.2 h after dark onset, suggesting that, in addition to the decrease in the total number of phagosomes shed associated with loss of *kif17*, the kinetics of disc shedding at night might also be altered. Additionally, the kinetics of phagosome digestion could be roughly estimated by the decreasing slope in the number of phagosomes observed following either the morning or night peak. However, this is under the assumption that there are no new phagosomes being shed during this period. Following the morning peak of disc shedding, we estimated similar slopes of 8 × 10^− 3^ and 10X10^− 3^ phagosomes/μm of RPE per hour in wild-type and *kif17*^*mw405*^ larvae respectively. Again, after the evening peak we estimated similar slopes of 9 × 10^− 3^ and 8 × 10^− 3^ phagosomes/μm of RPE per hour for wild-type and *kif17*^*mw405*^ larvae respectively. Taken together, these data suggest that the half-life of a phagosome of approximately 3–4 h is unaffected by the loss of *kif17.*

In addition to the deficiencies in disc shedding caused by loss of *kif17* in zebrafish, we observed a similar diminution in total number of phagosomes in *Kif17* deficient mice (Fig. 5d) without a significant impact on phagosome size (Fig. 5e). In contrast to the report that rat disc shedding occurs in only a single peak following light onset [28], we observed two peaks of disc shedding in mice, more comparable to our findings in zebrafish: one shortly after light onset and another shortly after dark onset. We further performed spline interpolation on both the wild-type and *Kif17*^*tm1b(Bjc)*^ phagosome number data (Additional file 1: Figure S9B) and estimated that the morning peak in phagosome number occurred 1.4 h after light onset in wild-type compared to 0.7 h in *Kif17*^*tm1b(Bjc)*^ mice, while the evening peak was estimated to occur at about 1.7 h after dark onset for both genotypes (Additional file 1: Table S2). Following the morning peak of disc shedding, phagosome number decreased at similar rates of 9 × 10^− 3^ and 11 × 10^− 3^ phagosomes/μm of RPE per hour in wild-type mice compared *Kif17*^*tm1b(Bjc)*^ mice. After the evening peak, the number of phagosomes decreased at similar rates of 6 × 10^− 3^ and 7 × 10^− 3^ phagosomes/μm of RPE per hour in wild-type and *Kif17*^*tm1b(Bjc)*^ mice respectively. Under all conditions, the half-life of a phagosome in mice is approximately 1–2 h. As in zebrafish, this analysis suggests little if any alteration in the kinetics of phagosome accumulation or digestion from loss of *Kif17*. In both mice and zebrafish, loss of *Kif17* is associated with a decrease in the total number of phagosomes, supporting a role for KIF17 in promoting disc shedding in the mature photoreceptor.

It has been suggested in an analysis using goldfish [29] that morning and evening peaks reflect disc shedding by rods and cones respectively, although the two types of phagosomes cannot be readily distinguished after phagocytosis. To further define these two peaks of disc shedding in zebrafish, we performed the immunogold rhodopsin labeling of phagosomes in wild-type larvae at both the morning (ZT 1.5) and evening (ZT 15.5) peaks. While there was no change in the total number of phagosomes at either of these peaks, there was an increase in the number of unlabeled phagosomes at the evening peak, suggesting an increase in cone disc shedding (Fig. 5c). However, at least 25% of the phagosomes present at the night peak were still rod phagosomes. Additionally, we performed immunogold labeling with the cone-specific transducin-α antibody, and again found that while there was no change in the total number of phagosomes at either the morning or evening peak, the evening peak contains a higher ratio of cone to rod phagosomes than the morning peak (Additional file 1: Figure S8C). However, cone phagosomes still composed about 40% of the phagosomes in the morning peak, while unlabeled phagosomes compose about 40% of the evening peak. Overall, there are two peaks of phagosomes in zebrafish: one shortly after light onset with a slightly higher proportion of rod phagosomes and another peak occurring shortly after dark onset with a slightly higher proportion of cone phagosomes. These zebrafish data ultimately do not support the absolute distribution of cone shedding at night and rod shedding during the day as is commonly reported in the literature [29], but rather only a small bias of cone shedding at night and rod shedding during day. Additionally, we performed immunogold rhodopsin labeling of phagosomes in mice with B630, a mouse monoclonal anti-rhodopsin, at both the morning (ZT 1.5) and evening (ZT 13.5) peaks. At both peaks, the majority of phagosomes contained rhodopsin, although there was a small proportion of unlabeled phagosomes presumably from cones (Fig. 5f). Of note, there was no significant increase in the number of these unlabeled phagosomes at the night-time peak.

### Constitutively active CaMKII promotes disc shedding in a Kif17 dependent manner

**Fig. 6:**
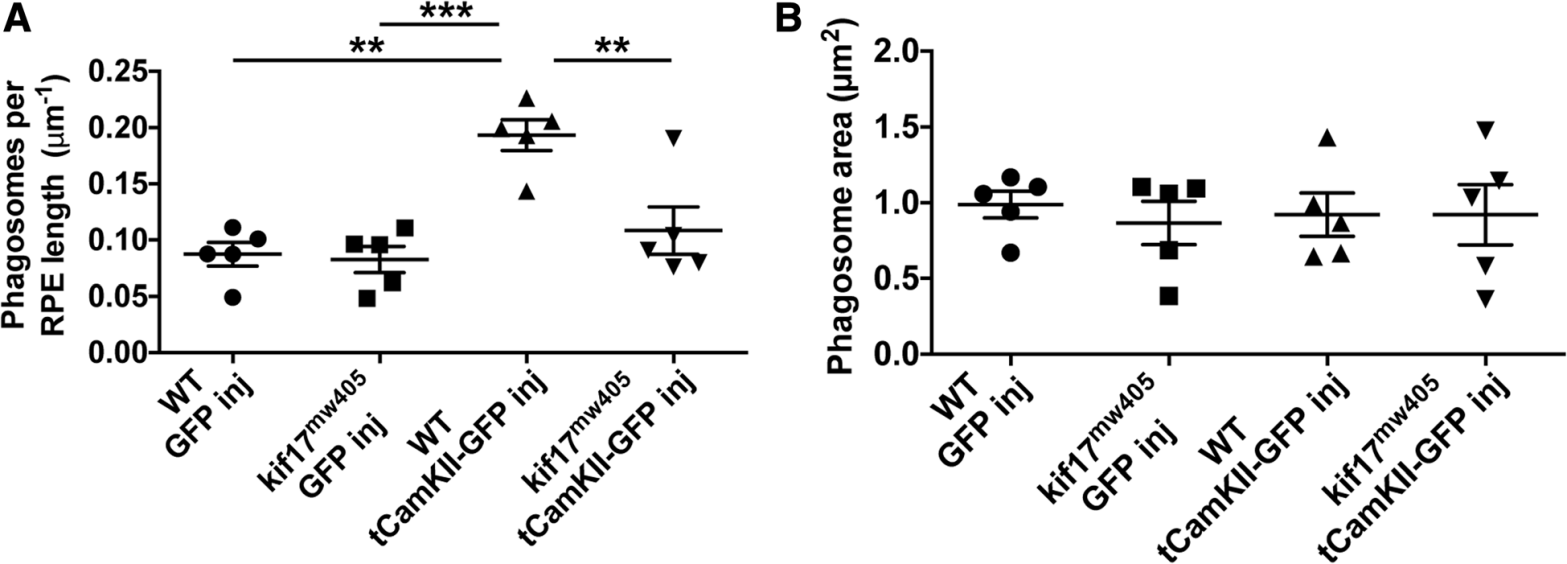
CaMKII promotes *kif17*-dependent disc shedding. **a** TEM phagosome number analysis of 7 dpf GFP-positive wild-type and *kif17*^*mw405*^ larvae injected with either control TaCP:GFP (*n* = 5 injected larvae, 843 μm of RPE for WT; *n* = 5, 795 μm of RPE for *kif17*^*mw405*^) or the constitutively active CaMKII construct TaCP:tCaMKII-GFP (*n* = 5, 1011 μm of RPE for WT; *n* = 5, 1322 μm of RPE for *kif17*^*mw405*^). **b** Quantification of the size of phagosomes for wild-type and *kif17*^*mw405*^ larvae injected with either TaCP:GFP (*n* = 5 injected larvae, 74 phagosomes for WT; *n* = 5, 70 phagosomes for *kif17*^*mw405*^) or TaCP:tCaMKII-GFP (*n* = 5, 193 phagosomes for WT; *n* = 5, 141 phagosomes for *kif17*^*mw405*^)

While we have shown that amino acid changes at the conserved zebrafish S815 phosphorylation site can control KIF17 localization and promote photoreceptor disc shedding, we sought to associate this regulation more directly to CaMKII, which phosphorylates this conserved serine of KIF17 in neurons [16] and is expressed in cones [30]. We injected tCaMKII-GFP, a constitutively active form of CaMKII [31], under control of the TaCP cone promoter and investigated disc shedding at 7dpf as compared to control larvae injected with soluble GFP under control of the same cone promoter. We found that wild-type larvae injected with tCaMKII-GFP had a two-fold increase in phagosome number as compared to soluble GFP controls. However, this increase in disc shedding did not occur when tCaMKII-GFP was injected in *kif17*^*mw405*^ larvae (Fig. 6a). There was no change in phagosome size associated with any of the injected larvae (Fig. 6b). Ultimately, these data show that the effect of tCaMKII depends on the presence of endogenous Kif17 and implicates CaMKII mediated phosphorylation of Kif17 in the regulation of disc shedding.

## Discussion

### Phosphorylation promotes ciliary localization of KIF17

In this work, we show that phospho-mimetic Kif17 is uniformly distributed along the length of the zebrafish cone photoreceptor OS compared to phospho-deficient Kif17, which appears to accumulate specifically in the proximal OS. Given the previous work suggesting that the NLS of KIF17 can regulate its ciliary entry through a classical nuclear import mechanism [8, 9], our original hypothesis was that phosphorylation of Kif17 via CaMKII could promote the OS entry of Kif17. However, phospho-deficient Kif17 does not appear to be entirely restricted from entering the OS, suggesting that this is not the mechanism by which phosphorylation regulates OS localization of Kif17. Instead, phosphorylation appears to promote localization towards the distal OS. Additionally, wild-type and phospho-deficient KIF17 both appear to have a comparable, basal level of ciliary entry when expressed in cultured mammalian cells, further suggesting that phosphorylation instead promotes ciliary accumulation rather than ciliary entry. One possible mechanism of this accumulation could be through an interaction with a plus-end microtubule associated protein. Kif17 has a consensus sequence for EB1-binding and microtubule plus-end accumulation [32] and has been shown to interact with EB1 in epithelial cells for regulating microtubule dynamics at the plus-ends of cytoplasmic microtubules [33]. EB1 also localizes to the axonemal tip of flagella in *Chlamydomonas reinhardtii* [34]. Thus, enhanced affinity for EB1 (or another related microtubule associated protein) via phosphorylation of Kif17 could lead to the enhanced distribution of Kif17 in the distal OS compared to non-phosphorylated Kif17 which appears to accumulate only in the proximal OS. De-phosphorylation could subsequently release KIF17 from this interaction, leading to removal from the cilia via either active transport or diffusion. In support of this model, truncated KIF17 lacking the motor domain is still able to accumulate at the ciliary tip [6, 9, 10], implying the need for an additional microtubule-associated protein for KIF17 ciliary localization. As an alternative mechanism, non-phosphorylated Kif17 could have reduced motility, preventing its normal trafficking along the axoneme and resulting in its accumulation at the proximal OS. KIF17 has been shown to undergo autoinhibition whereby the C-terminal tail folds and inhibits the motor domain [35], and it is possible that phosphorylation prevents this autoinhibited state. Future work focusing on the kinetics of phospho-mutant Kif17 trafficking through the OS and potential interacting partners will determine the specific mechanism by which phosphorylation can regulate the OS localization of Kif17.

In support of the finding that phosphorylation of Kif17 promotes its ciliary localization in zebrafish photoreceptors, we also find that phospho-mimetic KIF17 has enhanced ciliary localization in four different mammalian cell lines. In contrast, there was only a mild, cell line-dependent restriction of nuclear localization associated with phospho-mimetic KIF17. There are two explanations for these data: that KIF17 phosphorylation can directly inhibit nuclear entry only in certain cellular contexts or that the mild effects on nuclear entry are secondary effects of the strong promotion of ciliary localization. However, because all four cell lines depict a strong promotion of ciliary localization of phospho-mimetic KIF17, it is unlikely that the effects on nuclear localization seen in only two of the cell lines are secondary effects. Ultimately, this evidence suggests that KIF17 phosphorylation is a robust regulator of ciliary localization in a variety of cell types, including photoreceptors, and in a variety of species, with much milder effects on nuclear localization.

### KIF17 positively regulates disc shedding

The function of Kif17 in zebrafish photoreceptors has been ambiguous. While early morpholino knockdown experiments suggested that Kif17 was important for OS development [4], two genetic mutants [36, 37] were shown to have morphologically normal OS. Similarly, mice were shown to have morphologically normal photoreceptors [6]. We recently resolved these apparent contradictions by showing that loss of Kif17 delays the onset of OS morphogenesis in both zebrafish and mice [5]. Nonetheless, OS in both species attain normal morphology and dimensions [5, 6], raising the question of whether Kif17 functions in a mature photoreceptor.

In this work, we find significant decreases in disc shedding in both *kif17*^*mw405*^ zebrafish and *Kif17*^*tm1b(Bjc)*^ mice. While disc shedding is not completely ablated with loss of *Kif17*, we have previously proposed that additional factors, such as kinesin-II or diffusion, can compensate for defects in trafficking in *Kif17* mutant photoreceptors [5]. Whether other factors compensate for loss of KIF17’s function in promoting disc shedding and whether attenuating such factors can lead to compound effects in *Kif17* mutants on disc shedding remains to be elucidated. Additionally, cone expression of transgenic phospho-mimetic Kif17 results in a cell-autonomous enhancement of disc shedding. CaMKII appears to be one of the kinases responsible for phosphorylation of Kif17 in photoreceptors in vivo, as cone expression of constitutively active tCaMKII promotes disc shedding in a *kif17*-dependent manner. However, additional investigation is required to identify other potential kinases or the phosphatase that may regulate removal of Kif17 from the OS. Ultimately, the phospho-mimetic Kif17 “gain of function” effects in promoting disc shedding combined with decreased disc shedding phenotype associated with genetic loss of Kif17 strongly implicate Kif17 in an important role regulating disc shedding at the level of the photoreceptor. Major advances have been made regarding the regulation of disc shedding, but most of that work has been at the level of phagocytosis by adjacent RPE cells [38–40]. However, PS exposure at the distal OS precedes shedding events [12], and these data on Kif17 further imply an active process in the ciliary OS tip that promotes detachment of the distal domain and subsequent phagocytosis.

Another significant conclusion from this work is that photoreceptors can exhibit considerable plasticity in the kinetics of ciliary OS turnover while still maintaining normal dimensions. It is well known that mouse rods shed approximately 10% of their length daily and maintain a steady-state length through compensating OS renewal [41]. In the *Kif17* mutant mice and zebrafish, diminished disc shedding is not accompanied by reduced size of the OS [5], suggesting an overall slower rate of renewal at the OS base. Likewise, the dimensions of the OS are unchanged in the “gain of function” experiments involving phospho-mimetic transgene expression, implying that enhanced disc shedding is compensated for by enhanced OS formation. While we propose that Kif17 is implicated in regulating disc shedding directly given this work, it is possible that Kif17 is involved in promoting new morphogenesis at the OS base and the increased disc shedding is a secondary effect to maintain a steady-state OS. Although the open disc conformation of cone photoreceptors complicates experiments, techniques involving autoradiography [42] and fluorescent [43] pulse-labels to study the dynamics of rod OS morphogenesis can be applied to investigate the changes in rates of new cone OS morphogenesis associated with either gain or loss of function of Kif17.

As little is known about the photoreceptor-derived signaling that regulates disc shedding, the specific mechanism by which Kif17 promotes disc shedding is unclear. However, as PS exposure at the distal OS precedes disc shedding [12], it is possible that Kif17 is involved in regulating the localization of a putative scramblase [44] or floppase [45] involved in mediating PS transport from the cytosolic to extracellular leaflet of the OS membrane. Alternatively, Kif17 localized to the distal OS could interact with various ligands expressed in the photoreceptor, such as SEMA4D, that are involved in regulating disc shedding via receptors in the RPE [46]. Future work will address potential interacting partners of Kif17 to reveal how localization at the distal OS promotes disc shedding.

### Temporal regulation of cone and rod disc shedding

Our investigation of Kif17 loss of function required analysis of disc shedding at multiple timepoints because photoreceptor turnover is well-known to occur on a diurnal schedule [28, 29, 47], which led to unexpected surprises in our analysis in both zebrafish and mice. First, it is generally thought that rods shed in the morning after light onset, while cones do so in the evening after dark onset [29, 48, 49]. However, this has been generally determined by counting phagosomes in the RPE where the differences between the two cell types are not apparent. Our new data using immunogold labeling specific for either rods (using the rhodopsin antibody) or cones (using the GNAT2 antibody) show that both rod and cone phagosomes are present at both day-time and night-time peaks in zebrafish. Our interpretation of these data is that both rods and cones shed discs at either time of day, although there is a slight preponderance of rod phagosomes in the morning and cones at night. At the very least, the absolute idea of “rods shed during the day and cones at night” [29] must be reconsidered. Secondly, we observed equal peaks of disc shedding in mice both after light onset and offset, which contrasts with the view that disc shedding in rod-dominated rodents occurs principally after light onset [28]. This can hardly be explained as a dichotomy of rod versus cone shedding as the mouse retina consists of ~ 97% rods and ~ 3% cones [50]. One key difference between our analysis and other studies is that we used EM to image phagosomes, whereas most other studies have used light microscopy [28, 46, 51–53], which may be less sensitive in the detection of phagosomes, although EM has been used previously without a detectable increase in phagosome accumulation after dark [54]. Another potential discrepancy between our results and the conventional notion of a single peak of disc shedding is that we analyzed disc shedding from mice collected at symmetric time points, including both 1.5 h after both light onset and dark onset, whereas many other studies analyzed mice collected at wider intervals [46, 53], possibly missing important events occurring after dark onset. Nonetheless, several disc shedding studies in mice of different backgrounds (including 129/Sv, C3H, and C57BL/6) included timepoints in darkness and did not reveal as large a peak (if any) in phagosome accumulation after dark onset as our analysis [52, 54]. Ultimately, it is conceivable that our findings are the result of either a mouse strain difference or even species difference as the original study of rhythmic disc shedding in rats did include time points shortly after dark onset [28]. This surprising discrepancy in mice is of potential significance in understanding disc shedding and OS turnover and is under current investigation.

## Conclusion

Overall, we show that phosphorylation of KIF17 enhances its localization along the length of the cone photoreceptor outer segment, where it promotes cell-autonomous disc shedding. As disc shedding has been predominantly studied within the retinal pigment epithelium, this work implicates photoreceptor-derived signaling in the underlying mechanisms of disc shedding.

## Methods

### Mouse and zebrafish husbandry

The animals used in these experiments were maintained in animal care facilities fully accredited by the American Association of Laboratory Animal Science, and used in accordance with the ARVO Resolution on the Use of Animals in Research, with applicable portions of the Animal Welfare Act and with the DHHS “Guide for the Care and Use of Laboratory Animals”. All experiments were approved and conducted in accordance with the Institutional Animal Care and Use Committee of the Medical College of Wisconsin. Mice were maintained in a pathogen-free barrier facility in cages of between 2 and 5 mice with access to dry food, water, and bedding material under 12 h:12 h light:dark cycle. We generated the *Kif17*^*tm1b(Bjc)*^ mouse line (EUCOMM) as described previously [5] on the C57BL/6j background. Adult ZDR zebrafish (Aquatica Tropicals) were maintained at 28.5 °C on an Aquatic Habitats recirculating filtered water system (Aquatic Habitats) in reverse-osmosis purified water supplemented with Instant Ocean salts (60 mg/L) on a 14 h:10 h light:dark cycle. Zebrafish were housed up to 30 fish per 10 L tank (Aquatic Habitats) and fed twice daily with a mixture of brine shrimp and dry food. Embryos were raised at 28.5 °C on a 14 h:10 h light:dark cycle. We generated the *kif17*^mw405^ zebrafish line as described previously [5]. For all experiments involving animals, a biological replicate is defined as a single mouse or single zebrafish. Male and female animals were randomly assigned to each experimental group. For both zebrafish and mice, littermate animals were used. Prior to experiments, zebrafish were euthanized with Tricaine (Sigma-Aldrich) and mice were euthanized with CO_2_ inhalation followed by cervical dislocation according to approved protocols.

### Cloning of constructs

A plasmid encoding CMV:KIF17-mCherry (GenBank MK046074) was generated as previously reported [7]. Overlap extension PCR was performed to create the two mouse phospho-mutants: CMV:KIF17(S1029D)-mCherry (GenBank MK046075) and CMV:KIF17(S1029A)-mCherry (GenBank MK046076). The wild-type codon sequence for S1029, AGC, was mutated to GAC for S1029D and GCC for S1029A. A plasmid encoding TaCP:Kif17-GFP (GenBank MK046077) was generated as previously reported [7]. Overlap extension PCR was performed to create the two zebrafish phospho-mutants: TaCP:Kif17(S815D)-GFP (GenBank MK046078) and TaCP:Kif17(S815A)-GFP (GenBank MK046079). The wild-type codon sequence for S815, AGC, was mutated to GAC for S815D and GCC for S815A. The constitutively active CaMKII (tCaMKII-GFP) was generated by Yasunori Hayashi [31]. tCaMKII-GFP was cloned downstream of the TaCP promoter (GenBank MK046080) using the Gateway Cloning system (Invitrogen). The control TaCP:GFP plasmid was generated by Breandán Kennedy [55]. Full sequences of newly generated constructs are included in Additional files 2, 3, 4, 5, 6, 7, 8 (Plasmid Maps).

### Zebrafish transgenesis

For various experiments involving injected larvae, 9.2 nL of a working solution containing 25 ng/μL of a plasmid (encoding either TaCP:Kif17-GFP, TaCP:Kif17(S815D)-GFP, TaCP:Kif17(S815A)-GFP, TaCP:tCaMKII-GFP, or TaCP:GFP), as well as 0.05% phenol red, were injected into ZDR embryos at the 1-cell stage. For stable line generation of TaCP:Kif17-GFP, TaCP:Kif17(S815D)-GFP, and TaCP:Kif17(S815A)-GFP, 10 ng/μL transposase RNA was included in the injection solution to facilitate genome integration [25]. Injected embryos were subsequently raised and outcrossed to screen for germ-line transmission. GFP-positive embryos were raised and outcrossed for several generations to establish stable lines of low-expressing, single insert transgenic fish to minimize non-specific effects due to high levels of over-expression (Fig. 3a,b). For experiments, embryos were imaged with epifluorescence at 4dpf and fluorescence intensity of eyes was quantified to ensure comparable expression levels among the three transgenes (Fig. 3a,c).

### Antibodies

Commercial primary antibodies included: 6-11B-1, or mouse monoclonal anti-acetylated α-tubulin (Sigma), rabbit polyclonal anti-GNAT2 (MBL International). The rabbit polyclonal anti-green and blue cone opsin antibodies were gifts from Dr. Thomas Vihtelic (University of Notre Dame) [56]. The mouse monoclonal anti-rhodopsin antibodies K62-171c and B630 were gifts from Dr. Paul Hargrave (University of Florida) [27]. Nuclei were stained with Hoechst (Thermo Scientific). Fluorescent secondary antibodies included goat anti-mouse IgG Alexa Fluor 488 and goat anti-rabbit IgG Alexa Fluor 594. Immunogold labeling secondary was either goat anti-mouse IgG 10 nm colloidal gold (Electron Microscopy Sciences) or goat anti-rabbit IgG 10 nm colloidal gold (Electron Microscopy Sciences).

### Mammalian cell culture analysis

LLC-PK1 (ATCC CL-101), HEK-293 (ATCC CRL-1573), hTERT-RPE1 (ATCC CRL-4000), and IMCD3 (ATCC CRL-2123) were obtained from ATCC. Cell line authentication and testing of mycoplasma-negative were performed by ATCC. Additional testing of mycoplasma infection is performed biannually with MycoAlert (Lonza). LLC-PK1 cells were grown in Medium 199 (Gibco), HEK-293 cells were grown in DMEM (Gibco), and hTERT-RPE1 and IMCD3 cells were grown in DMEM/F12 (1:1) (Gibco). All media were supplemented with 10% FBS and 1% penicillin and streptomycin and cells were grown at 37 °C in 5% CO_2_. For ciliogenesis and transfection, cells were transferred to Opti-MEM (Gibco) at 60–80% confluency and transiently transfected with Lipofectamine 3000 (Invitrogen). Serum-starvation in Opti-MEM induced ciliogenesis and after 24 h, cells were fixed in 4% PFA for 20 min and subsequently immunostained with acetylated α-tubulin to indicate cilia and Hoechst to label nuclei. Because of the nature of transient transfection, there were a variety of transgenic expression levels. Approximately 20% of cells with a high level of expression in which transgene appeared to be highly vesicular or stuck in Golgi were excluded from analysis. For analysis, five separate transfections of each transgene in each cell line were performed. For ciliary localization in each cell line, the number of cells with mCherry labeling of the cilia was counted and divided by the total number of transfected cells. For nuclear localization, ImageJ was used to quantify the average intensity of both cytoplasmic and nuclear mCherry signal. Following subtraction of the average intensity of the background of each image (quantified from an area where there was no cellular fluorescence), the ratio of the cytoplasmic to nuclear average intensity was calculated.

### Photoreceptor localization analysis

Embryos were injected with constructs as described above for mosaic expression and raised in phenylthiourea (PTU) to delay melanin pigment formation. At 4 dpf, larvae were anesthetized with tricaine and screened for GFP fluorescence. Immunofluorescence staining was performed on cryosections of 5 dpf GFP-positive larvae as previously described [4] with antibodies to green or blue opsin to label cone OS and Hoechst to label nuclei. For quantification, only injected larvae with positive transgenic expression were analyzed. For OS localization, GFP labeling in the opsin-labeled OS was analyzed compared to the total number of transgene-expressing cones. For line intensity analysis, a line along the length of the OS (as determined by opsin-labeling) was used to calculate GFP signal intensity in ImageJ. For standardization of levels of transgene expression, all intensity values along a single line were standardized to the maximum value along that line. Additionally, to standardize for variations in lengths of the OS, the length of an OS was split into ten equal groups based on position from base to distal OS and intensity values were averaged for each of these ten groups to generate data for a single technical replicate. In each biological replicate, several technical replicates were averaged, as described in captions.

### Disc shedding analysis

For transgenic or injected larva disc shedding analyses, zebrafish embryos were raised in phenylthiourea until 4 dpf when they could be screened for GFP fluorescence. GFP-positive embryos were then raised in regular fish water until either 5dpf (Additional file 1: Figure S5) or 7dpf (rest of the experiments), when they were collected at 1.5 h following light onset. For zebrafish diurnal disc shedding analysis, 14dpf wild-type and *kif17*^*mw405*^ larvae were collected at nine different timepoints following light onset: 0 (light onset), 1.5 h, 3.5 h, 7.5 h, 10.5 h, 14 h (dark), 15.5 h, 17.5 h, 20.5 h. Collected larvae were processed as previously described for TEM [5]. For mouse diurnal disc shedding analysis, 2- to 4-month old C57BL/6j and *Kif17*^*tm1b(Bjc)*^ litter-mate mice were collected at eight different timepoints following light onset: 0 (light onset), 1.5 h, 4.0 h, 8.0 h, 12 h (dark), 13.5 h, 16 h, and 20 h. Mice were euthanized and whole eyes were processed as previously described for TEM [5]. For zebrafish analysis, five larvae were analyzed for each genotype and timepoint for a total of 45 larvae per genotype. For mouse analysis, three mice were analyzed for each genotype and timepoint for a total of 24 mice per genotype. For phagosome number analysis, the number of phagosomes was counted and divided by the total length of RPE measured. For phagosome size analysis, phagosome area was measured and averaged for each sample. For OS size analysis, OS area was measured and averaged for each sample.

### Spline interpolation analysis

For zebrafish, cubic spline interpolation was performed in Prism 6 (GraphPad) with 40 segments over the 24 h period (including the ZT 0 timepoint repeated at ZT 24) for increments of 0.615385 h. For mice, cubic spline interpolation was performed with 36 segments for increments of 0.6857143 h. For analysis, the maximum disc shedding rate during both the morning and evening was determined as the peak of disc shedding. To calculate the digestion rates of phagosomes, the maximum and minimum points of disc shedding for both the morning and evening shedding events was determined. Rates of phagosome digestion were subsequently calculated as the decreasing slope of the number of phagosomes over the period of between 25 and 75% of the total time between maximum and minimum. Phagosome half-life was calculated for both morning and evening as the time after the maximum peak until the phagosome number reached half-way between the maximum and minimum points.

### Immunogold labeling

For immunogold analysis, 7dpf transgenic larvae were collected at 1.5 h after light onset or 14dpf wild-type larvae were collected at both 1.5 h after light onset and 1.5 h after dark onset. Larvae were fixed overnight in 0.1% glutaraldehyde and 2% paraformaldehyde in 0.1 M phosphate buffer (pH 7.4). Larvae were washed in phosphate buffer, dehydrated with two 30 min washes of 70% ethanol, and incubated with a 2:1 mixture of LR white:70% ethanol for 1 h. Larvae were then incubated overnight in LR white at room temperature. In the morning, following two 30 min changes of LR white, larvae were embedded and polymerized at 55 °C for 48 h. Ultra-thin sections were mounted on 200 mesh formvar/carbon coated copper grids. Grids were incubated on drops of 0.15 mM glycine for 10 min, then on 5% BSA in 0.1 M phosphate buffer for 15 min. For labeling, grids were incubated on drops of antibody for 90 min. Grids were washed in 5% BSA in 0.1 M phosphate buffer and incubated with colloidal gold secondary (Electron Microscopy Sciences) for 1 h. Grids were washed in double-distilled H_2_O and stained with 1% aqueous uranyl acetate for 2 min. For each analysis, a total of five larvae were analyzed for each condition. For analysis, the number of both gold-labeled and unlabeled phagosomes was counted and divided by the total length of RPE measured.

### qPCR analysis

For mouse circadian *Kif17* expression analysis, adult C57BL/6 mouse retinas were collected every four hours beginning at light onset over a 24 h period and placed RNAlater (Ambion) overnight at 4 °C. For zebrafish circadian *kif17* expression analysis, 14dpf ZDR zebrafish eyes were collected at nine discrete timepoints following light onset: 0 (light onset), 1.5 h, 3.5 h, 7.5 h, 10.5 h, 14 h (dark), 15.5 h, 17.5 h, 20.5 h. For each biological replicate, three zebrafish eyes were pooled. For zebrafish opsin expression analysis, five whole 7dpf zebrafish larvae were pooled. Following sample collection, RNA was extracted, cDNA synthesized, and qPCR run as previously described [5]. For each data-point, three biological replicates were run in triplicate for both mouse and zebrafish samples. For mouse, published *Kif17* primers were used [57] and published RNA polymerase II primers were used as a reference gene [58]. For zebrafish, published ef1*α* primers were used as a reference gene [5], published opsin primers were used [5], and *kif17* primers are depicted below.

**Table:** 

Primer	Sequence	Efficiency
*kif17* (F)	GGATGAAGAGGGAGGAAC	98.5%
*kif17* (R)	CAATGACACCAGAAGGAG	

All expression values were standardized to 1 for the light onset timepoint (ZT 0).

### Imaging

Immunofluorescent images were acquired with a 100x, 1.30 NA oil-immersion lens (Nikon) on either an Eclipse TE300 (Nikon) microscope operating NIS-Elements (Nikon) and a Zyla sCMOS camera (Andor) or a C1 Plus-EX3 AOM Confocal System (Nikon) operating EZ-C1 (Nikon). TEM imaging was performed on a Hitachi H-600 with an ORCA-100 digital camera (Hamamatsu). Blinded image analysis was performed on de-identified, randomized images with ImageJ.

### Statistical analysis

For ciliary and nuclear localization of KIF17 in mammalian cell culture experiments, a one-way ANOVA was performed. Following significance, a Bonferroni multiple-comparisons post-hoc test was performed to determine significance between groups. For OS localization frequency and nuclear localization in zebrafish photoreceptors, a one-way ANOVA was performed. For line intensity analysis, a two-way ANOVA was performed. For single-timepoint disc shedding analysis in transgenic or injected larvae, a one-way ANOVA was performed. For immunogold phagosomes analysis in either transgenic or wild-type larvae, a two-way ANOVA was performed. For circadian disc shedding analysis in either zebrafish or mice, a two-way ANOVA was performed. For circadian *Kif17* mRNA expression in either mouse or zebrafish retinas, a one-way ANOVA was performed. For qPCR analysis of opsin expression, a one-way ANOVA was performed for each opsin gene. For all experiments, following significance with either one-way or two-way ANOVA, a Bonferroni multiple-comparisons post-hoc test was performed to determine significance between groups. For all experiments, values are expressed as mean ± SEM and significance is labeled as *: *p* ≤ .05, **: *p* ≤ .01, ***: *p* ≤ .001, ****: *p* ≤ .0001. Non-significant values are unlabeled.

## Additional files

Additional file 1: **Figure S1.** Phospho-mutations of S1029 regulate ciliary localization of KIF17. **Figure S2.** Phospho-mutations of S1029 mildly regulate nuclear localization of KIF17. **Figure S3.** Phospho-mutations of S815 regulate photoreceptor OS localization of Kif17. **Figure S4.** Mouse and zebrafish *Kif17* are expressed rhythmically. **Figure S5.** Transient, episomal expression of phospho-mimetic Kif17(S815D) increases disc shedding. **Figure S6.** Opsin expression is largely unaffected by transgene expression. **Figure S7.** Rhodopsin immunogold labeling of phagosomes. **Figure S8.** Cone transducin-α immunogold labeling of phagosomes**. Figure S9.** Spline interpolation of zebrafish disc shedding data. **Table S1.** Spline interpolation of zebrafish disc shedding. **Table S2.** Spline interpolation of mouse disc shedding. (DOCX 5111 kb)

Additional file 2: Plasmid map of CMV:KIF17-mCherry in GenBank file format (GB 17 kb)

Additional file 3: Plasmid map of CMV:KIF17(S1029D)-mCherry in GenBank file format (GB 17 kb)

Additional file 4: Plasmid map of CMV:KIF17(S1029A)-mCherry in GenBank file format (GB 17 kb)

Additional file 5: Plasmid map of TaCP:Kif17-GFP in GenBank file format (GB 22 kb)

Additional file 6: Plasmid map of TaCP:Kif17(S815D)-GFP in GenBank file format (GB 21 kb)

Additional file 7: Plasmid map of TaCP:Kif17(S815A)-GFP in GenBank file format (GB 21 kb)

Additional file 8: Plasmid map of TaCP:tCaMKII-GFP in GenBank file format (GB 19 kb)

## Abbreviations

COS: Cone outer segment
NLS: Nuclear localization signal
OS: Outer segment
PS: Phosphatidylserine
RPE: Retinal pigment epithelium
ZT: Zeitgeber Time
