# Supplementary material for: Kif17 phosphorylation regulates photoreceptor outer segment turnover
Source: BMC Cell Biol. 2018 Nov 20;19:25. doi: 10.1186/s12860-018-0177-9 (PMC6245759; doi:10.1186/s12860-018-0177-9)
Supplement: Supplementary file 1 — Figure S1. Phospho-mutations of S1029 regulate ciliary localization of KIF17. Figure S2. Phospho-mutations of S1029 mildly regulate nuclear localization of KIF17. Figure S3. Phospho-mutations of S815 regulate photoreceptor OS localization of Kif17. Figure S4. Mouse and zebrafish Kif17 are expressed rhythmically. Figure S5. Transient, episomal expression of phospho-mimetic Kif17(S815D) increases disc shedding. Figure S6. Opsin expression is largely unaffected by transgene expression. Figure S7. Rhodopsin immunogold labeling of phagosomes. Figure S8. Cone transducin-α immunogold labeling of phagosomes. Figure S9. Spline interpolation of zebrafish disc shedding data. Table S1. Spline interpolation of zebrafish disc shedding. Table S2. Spline interpolation of mouse disc shedding. (DOCX 5111 kb) [file 12860_2018_177_MOESM1_ESM.docx]

**
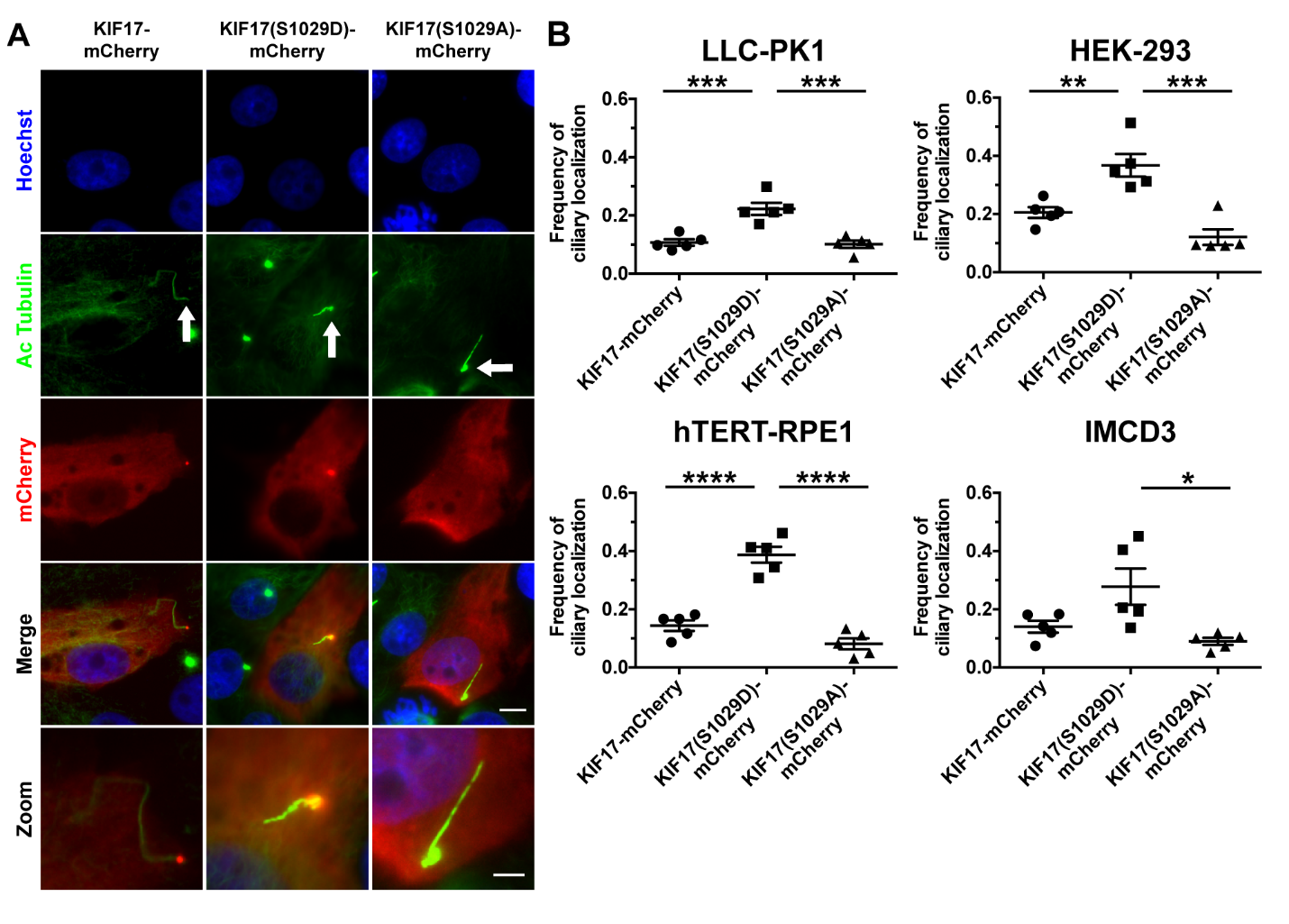
**

**Figure S1. Phospho-mutations of S1029 regulate ciliary localization of KIF17**

**(A)** LLC-PK1 cells were serum-starved to induce ciliogenesis and transfected with either KIF17-mCherry (left), KIF17(S1029D)-mCherry (middle), or KIF17(S1029A)-mCherry (right). 24 hours following transfection, cells were stained with Hoechst (blue) to label nuclei and acetylated α-tubulin (green) to label axonemes. The arrows mark the cilia. The zoom panel depicts a high-magnification of the cilium. Of note, transgenic KIF17 accumulates within the cilium (arrows), presumably at the ciliary tip as observed previously [8] [9] [6]. Cilia are often twisted and non-linear. Additionally, there is generally significantly less nuclear than cytoplasmic localization of each KIF17 transgene. Scale bar is 10 μm for normal magnification images. Scale bar is 4 μm for zoom panel. **(B)** Quantification of frequency of ciliary localization of each transgene 24 hours post-transfection in various mammalian ciliated cell lines. In LLC-PK1 cells, ciliary localization of KIF17-mCherry (n=5 transfections, 710 cells), KIF17(S1029D)-mCherry (n=5, 859 cells), and KIF17(S1029A)-mCherry (n=5, 664 cells) is shown. In HEK-293 cells, ciliary localization of KIF17-mCherry (n=5 transfections, 690 cells), KIF17(S1029D)-mCherry (n=5, 1149 cells), and KIF17(S1029A)-mCherry (n=5, 886 cells) is shown. In hTERT-RPE1 cells, ciliary localization of KIF17-mCherry (n=5 transfections, 280 cells), KIF17(S1029D)-mCherry (n=5, 307 cells), and KIF17(S1029A)-mCherry (n=5, 279 cells) is shown. In IMCD3 cells, ciliary localization of KIF17-mCherry (n=5 transfections, 665 cells), KIF17(S1029D)-mCherry (n=5, 815 cells), and KIF17(S1029A)-mCherry (n=5, 845 cells) is shown.


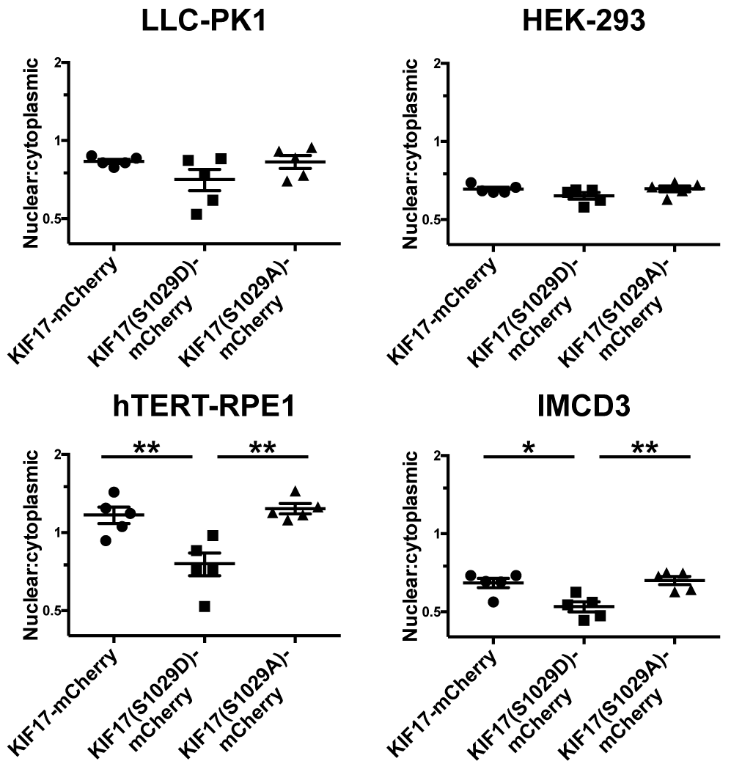


**Figure S2. Phospho-mutations of S1029 mildly regulate nuclear localization of KIF17**

Quantification of the ratio of nuclear to cytoplasmic intensity of each transgene 24 hours post-transfection in various mammalian ciliated cell lines. The ratio of nuclear to cytoplasmic intensity is depicted on a logarithmic scale. In LLC-PK1 cells, ratio of nuclear to cytoplasmic intensity of KIF17-mCherry (n=5, 268 cells), KIF17(S1029D)-mCherry (n=5, 260 cells), and KIF17(S1029A)-mCherry (n=5, 226 cells) is shown. In HEK-293 cells, ratio of nuclear to cytoplasmic intensity of KIF17-mCherry (n=5, 243 cells), KIF17(S1029D)-mCherry (n=5, 306 cells), and KIF17(S1029A)-mCherry (n=5, 337 cells) is shown. In hTERT-RPE1 cells, ratio of nuclear to cytoplasmic intensity of KIF17-mCherry (n=5, 198 cells), KIF17(S1029D)-mCherry (n=5, 182 cells), and KIF17(S1029A)-mCherry (n=5, 181 cells) is shown. In IMCD3 cells, ratio of nuclear to cytoplasmic intensity of KIF17-mCherry (n=5, 362 cells), KIF17(S1029D)-mCherry (n=5, 369 cells), and KIF17(S1029A)-mCherry (n=5, 525 cells) is shown. Of note, only in hTERT-RPE1 cells do the KIF17-mCherry and KIF17(S1029A)-mCherry transgenes both show greater localization in the nucleus compared to cytoplasm as indicated by a nuclear to cytoplasmic ratio greater than 1. In IMCD3 cells a similar difference is seen between Kif17(S1029D)-mCherry and the other two transgenes, but the nuclear to cytoplasmic ratio is less than one.


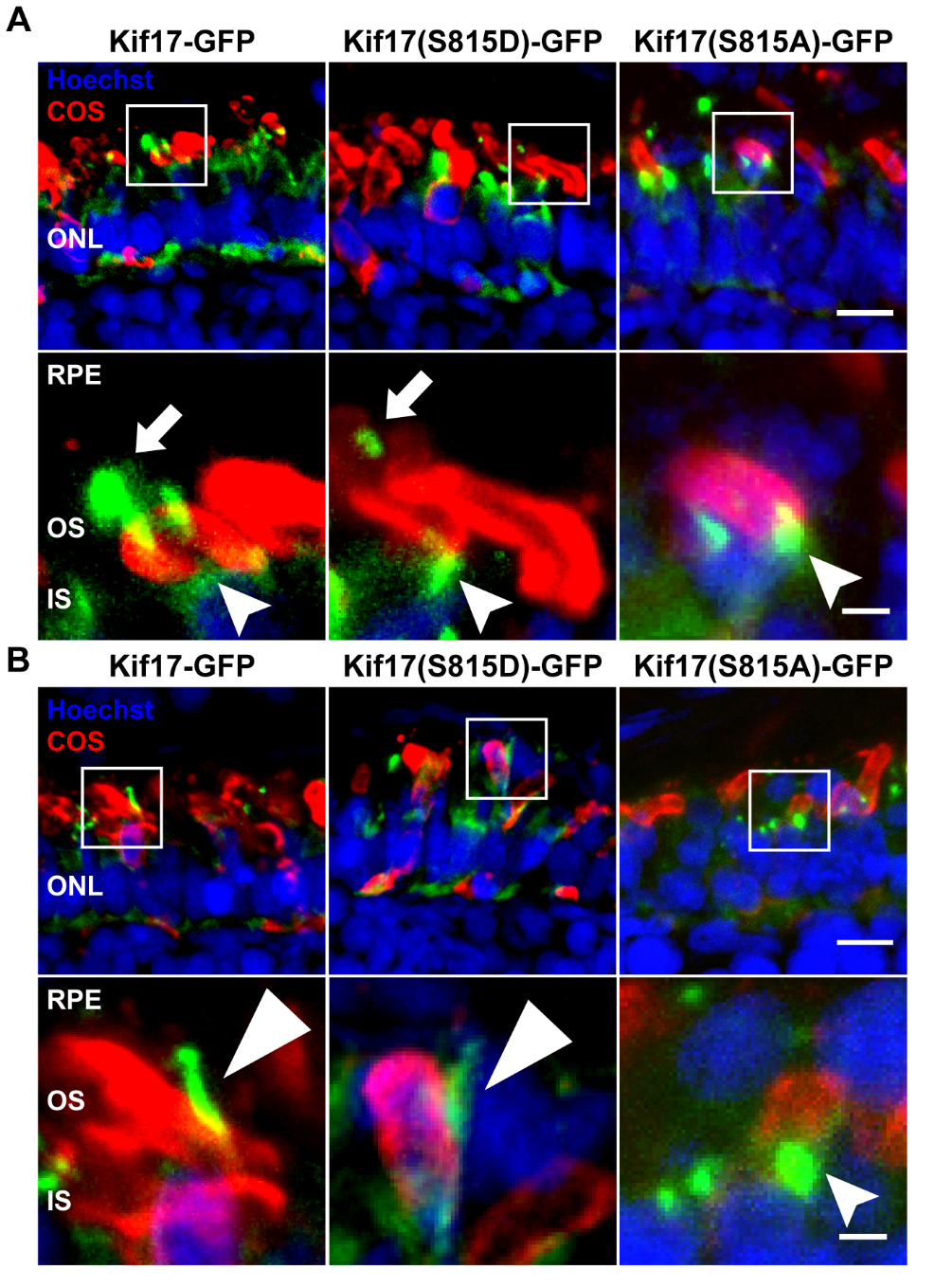


**Figure S3. Phospho-mutations of S815 regulate photoreceptor OS localization of Kif17**

**(A)** Related to **Figure 2,** 5 dpf larvae previously injected at the one cell stage with one of three different transgenic constructs under control of the TaCP promoter for expression in cone photoreceptors: Kif17-GFP (left), phospho-mimetic Kif17(S815D)-GFP (middle), and phospho-deficient Kif17(S815A)-GFP (right) were stained with Hoechst (blue) to label nuclei and blue cone opsin to label cone OS (COS, red). A z-series of confocal images was taken with 0.2 μm steps through the depth of the photoreceptor OS. Shown are projections of the z-series depicting the varying localization patterns of each of the three KIF17 transgenes. Both Kif17-GFP and phospho-mimetic Kif17(S815D)-GFP occasionally localize to the distal ends of cone OS. However, phospho-deficient Kif17(S815A)-GFP is observed accumulating at the base of the cone OS. Bottom panel is the indicated inset of the top panel. Scale bar is 5 μm for top panel, 1 μm for inset. **(B)** Both Kif17-GFP and phospho-mimetic Kif17(S815D)-GFP can also localize through the length of the axoneme along the side of the OS. Again, phospho-deficient Kif17(S815A)-GFP is observed accumulating at the base of the cone OS. Bottom panel is the indicated inset of the top panel. Scale bar is 5 μm for top panel, 1 μm for inset.


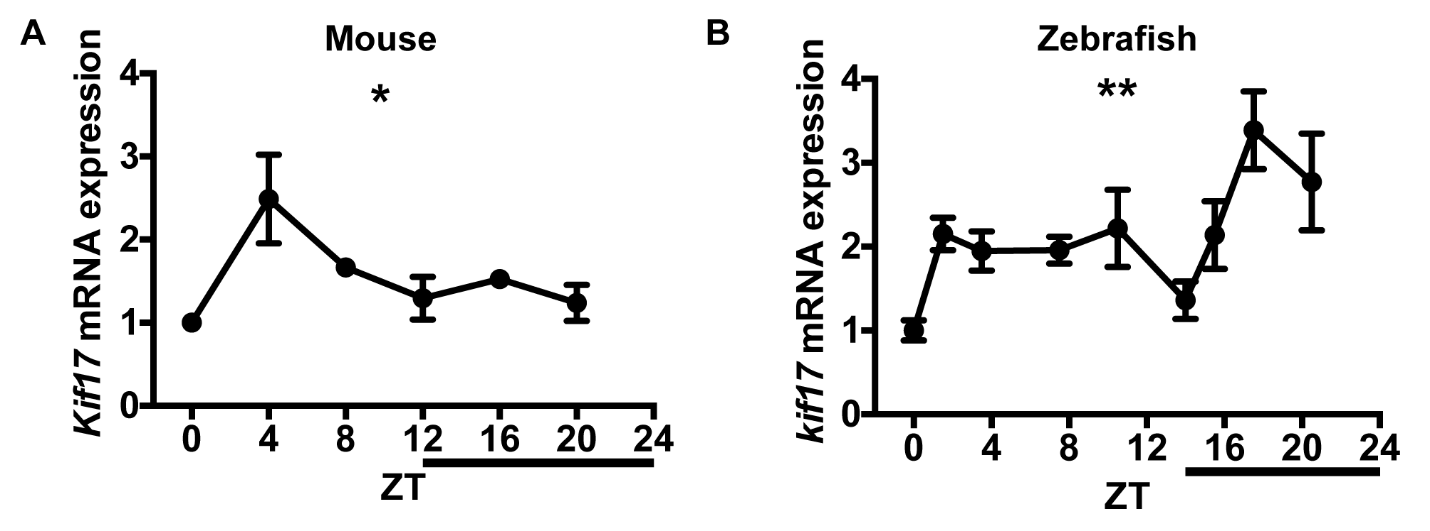


**Figure S4. Mouse and zebrafish *Kif17* are expressed rhythmically**

**(A)** Relative *Kif17* expression in adult mouse retinas that were collected every four hours following light onset (ZT 0). Three biological samples were analyzed at each timepoint with three technical replicates per biological sample. Dark onset for mice is at ZT 12 (black bar). One-way ANOVA was performed to determine statistical significance across all timepoints (p=0.0245, *). **(B)** Relative *kif17* expression in 14 dpf zebrafish eyes that were collected at nine discrete timepoints following light onset (ZT 0). Eyes from three 14 dpf zebrafish were pooled for a single biological sample, and three separate biological samples were analyzed at each timepoint with three technical replicates per sample. Dark onset for zebrafish is at ZT 14 (black bar). One-way ANOVA was performed to determine statistical significance in differences across all timepoints (p=0.0073, **).


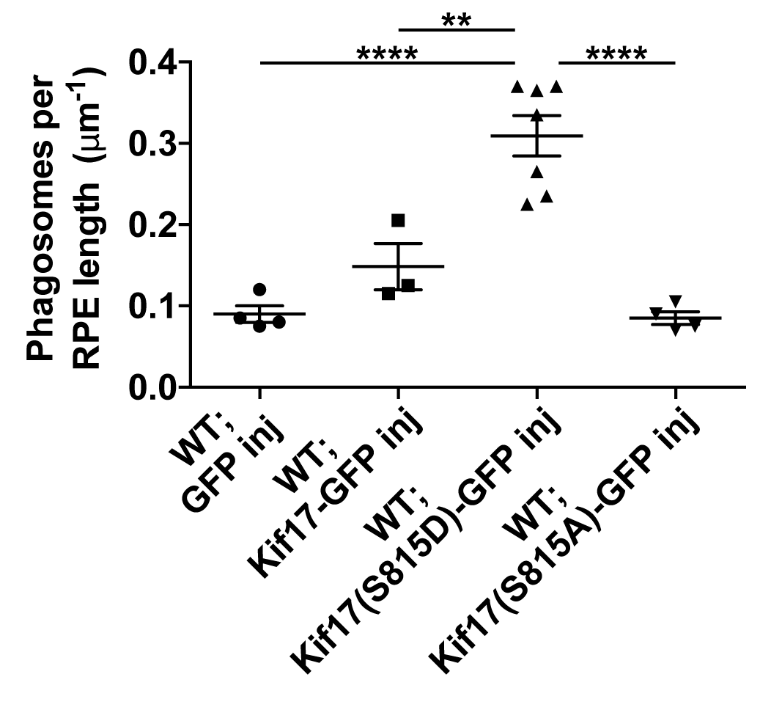


**Figure S5. Transient, episomal expression of phospho-mimetic Kif17(S815D) increases disc shedding**

Quantification of the number of phagosomes for 5dpf wild-type embryos injected with either TaCP:GFP (n=4 larvae, 800 μm of RPE), TaCP:Kif17-GFP (n=3, 600 μm of RPE), TaCP:Kif17(S815D)-GFP (n=7, 1400 μm of RPE), or TaCP:Kif17(S815A)-GFP (n=4, 800 μm of RPE). One-way ANOVA was performed to determine statistical significance in differences among injection groups (p<0.0001, ****). A post-hoc Bonferroni analysis to compare groups was performed to show a significant difference between Kif17(S815D)-GFP and: GFP (p<0.0001, ****), Kif17-GFP (p=0.0013, **), and Kif17(S815A)-GFP (p<0.0001, ****).


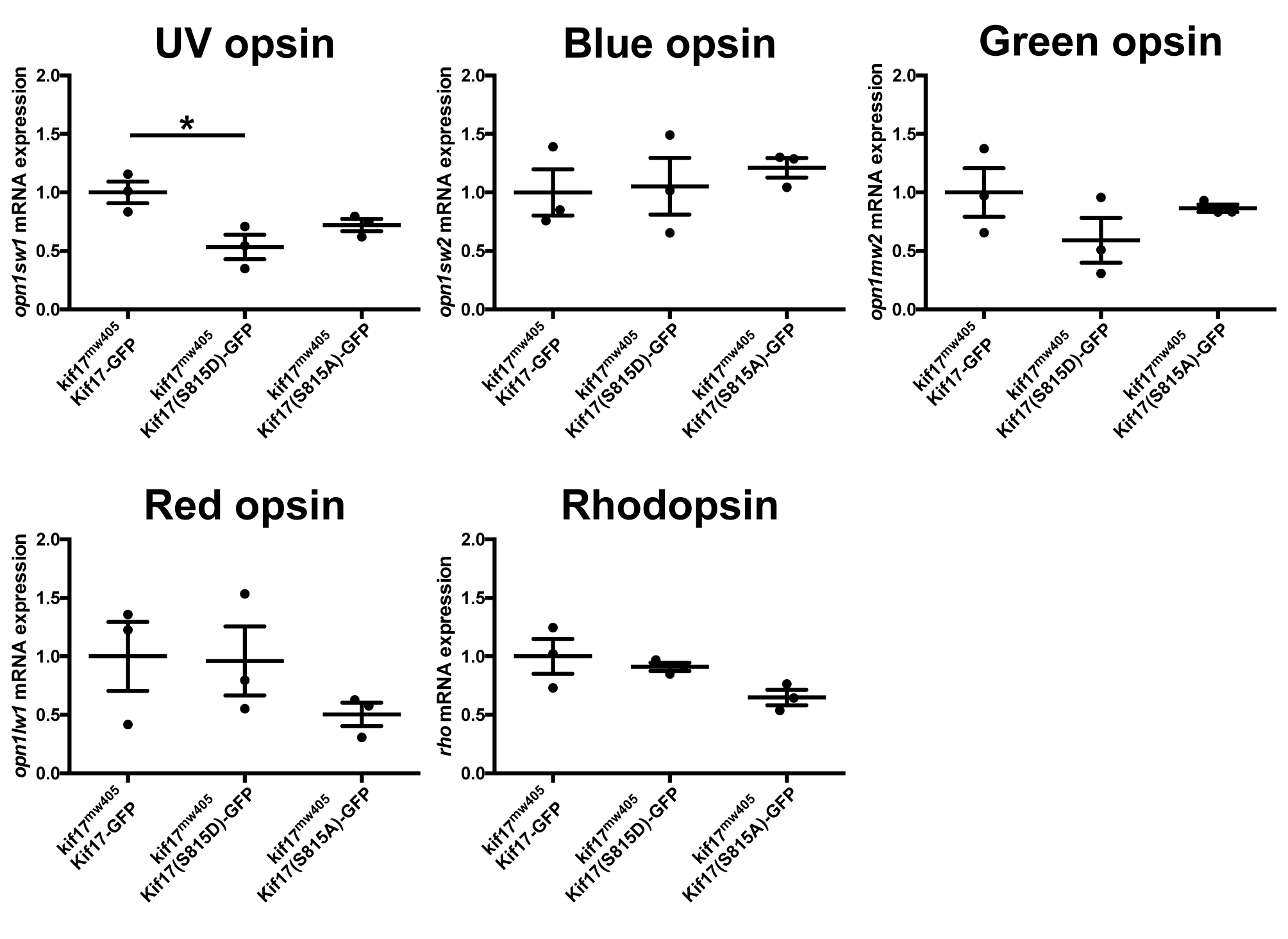


**Figure S6. Opsin expression is largely unaffected by transgene expression**

qPCR analysis of opsin expression for rods (rhodopsin) and cones (UV, blue, green, and red opsin) in 7dpf *kif17^mw405^* zebrafish. Five transgenic larvae were pooled for each sample. Three biological replicates were run with three technical replicates per biological sample. One-way ANOVA was performed for each opsin gene. A statistically significant difference between transgenes was only observed for UV opsin expression (p=0.0240, *). A post-hoc Bonferroni analysis to compare groups was performed to show a significant difference between Kif17-GFP and Kif17(S815D)-GFP (p=0.0261, *).

**
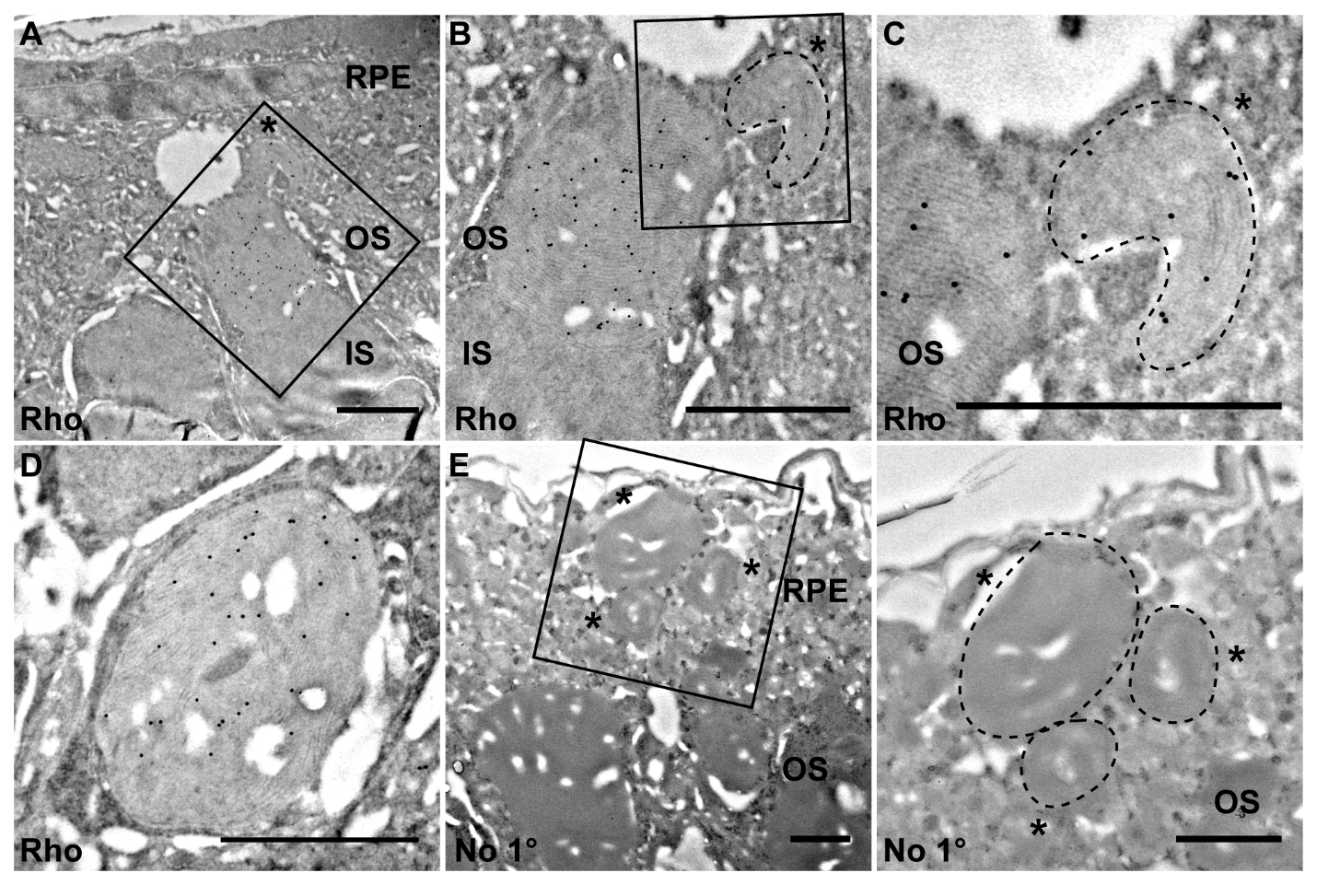
**

**Figure S7. Rhodopsin immunogold labeling of phagosomes**

**(A)** TEM image of a rod OS with a recently shed phagosome (*). Of note, the rod OS and phagosome are both labeled with the rhodopsin antibody K62-171c. Scale bar is 1 μm. **(B)** Inset of the squared region of **(A)** shows a higher magnification of the rod OS and the recently shed phagosome (*) that is outlined. Scale bar is 1 μm. **(C)** An additional inset of the squared region of **(B)** specifically shows the rod OS distal tip and the recently shed phagosome (*) that is outlined. Scale bar is 1 μm. **(D)** A separate TEM image showing the typical density of immunogold labeling of the rhodopsin antibody K62-171c in a separate phagosome. Scale bar is 1 μm. **(E)** TEM image of a rod OS with several phagosomes (*) that are a no primary, negative control for non-specific secondary immunogold labeling. Of note, no gold particles are observed at either the original magnification (left) or the high magnification inset (right). Scale bar is 1 μm.


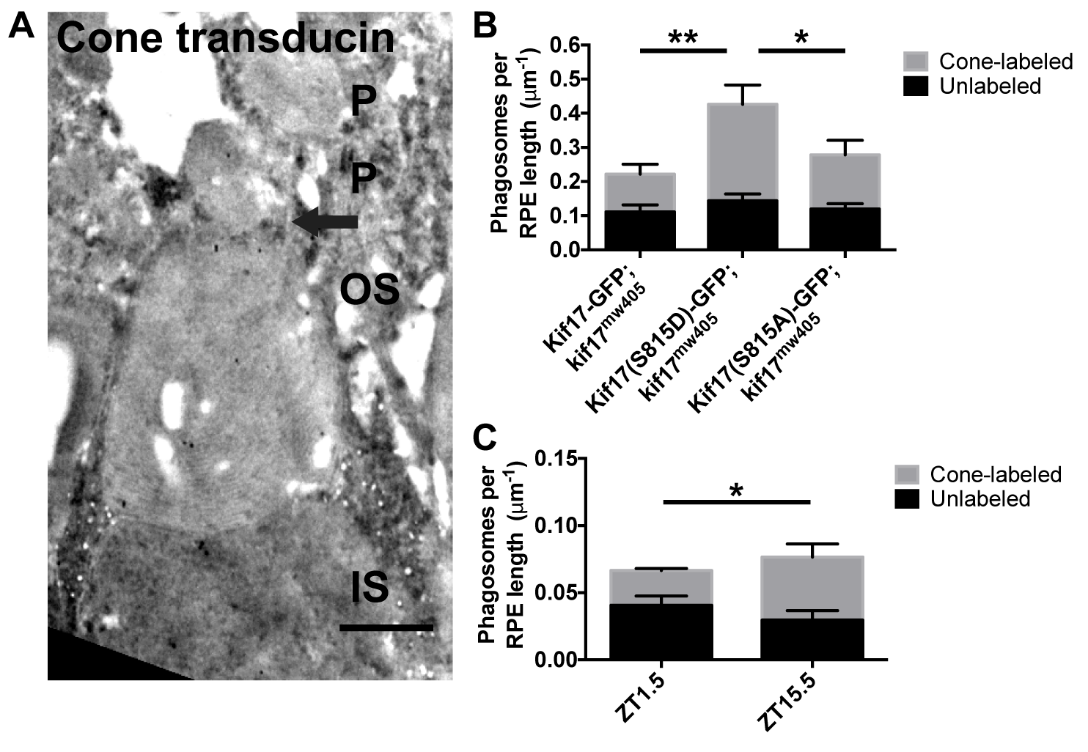


**Figure S8. Cone transducin-α immunogold labeling of phagosomes**

**(A)** TEM image of a cone OS with a recently shed phagosome (P). Of note, the cone OS and phagosome are both sparsely labeled with the cone transducin-α (gnat2) antibody, suggesting that while specific for cone photoreceptors, the immunogold labeling is rather inefficient. Scale bar is 0.5 μm. P is phagosome. **(B)** Analysis of immunogold labeling of cone transducin-α-containing or unlabeled phagosomes in 7 dpf Kif17-GFP, *kif17^mw405^* (n=5 larvae, 774 μm of RPE); Kif17(S815D)-GFP, *kif17^mw405^* (n=5, 1059 μm of RPE); and Kif17(S815A)-GFP, *kif17^mw405^* (n=5, 807 μm of RPE) larvae. Two-way ANOVA was performed to determine significance in the difference of the total number of phagosomes between transgenes (p=0.0172, *) and between the types of phagosomes (p=0.0434, *). Bonferroni’s multiple comparisons post-hoc test was performed to determine significance in the differences of cone transducin-α-labeled phagosomes between Kif17-GFP and Kif17(S815D)-GFP (p=0.0045, **) and between Kif17(S815A)-GFP and Kif17(S815D)-GFP (p=0.0485, *). There were no differences in numbers of unlabeled phagosomes between the transgenes. **(C)** Immunogold labeling of cone transducin-α-containing phagosomes in 14 dpf wild-type larvae at either the morning peak of disc shedding, ZT 1.5 (n=5 larvae, 924 μm of RPE), or the evening peak, ZT 15.5 (n=5, 1072 μm of RPE). Two-way ANOVA was performed to determine significance in the interaction between the time of day and the concentration of rod or cone phagosomes (p=0.0340, *). There were no statistical differences in the total number of phagosomes between the morning or evening peak.


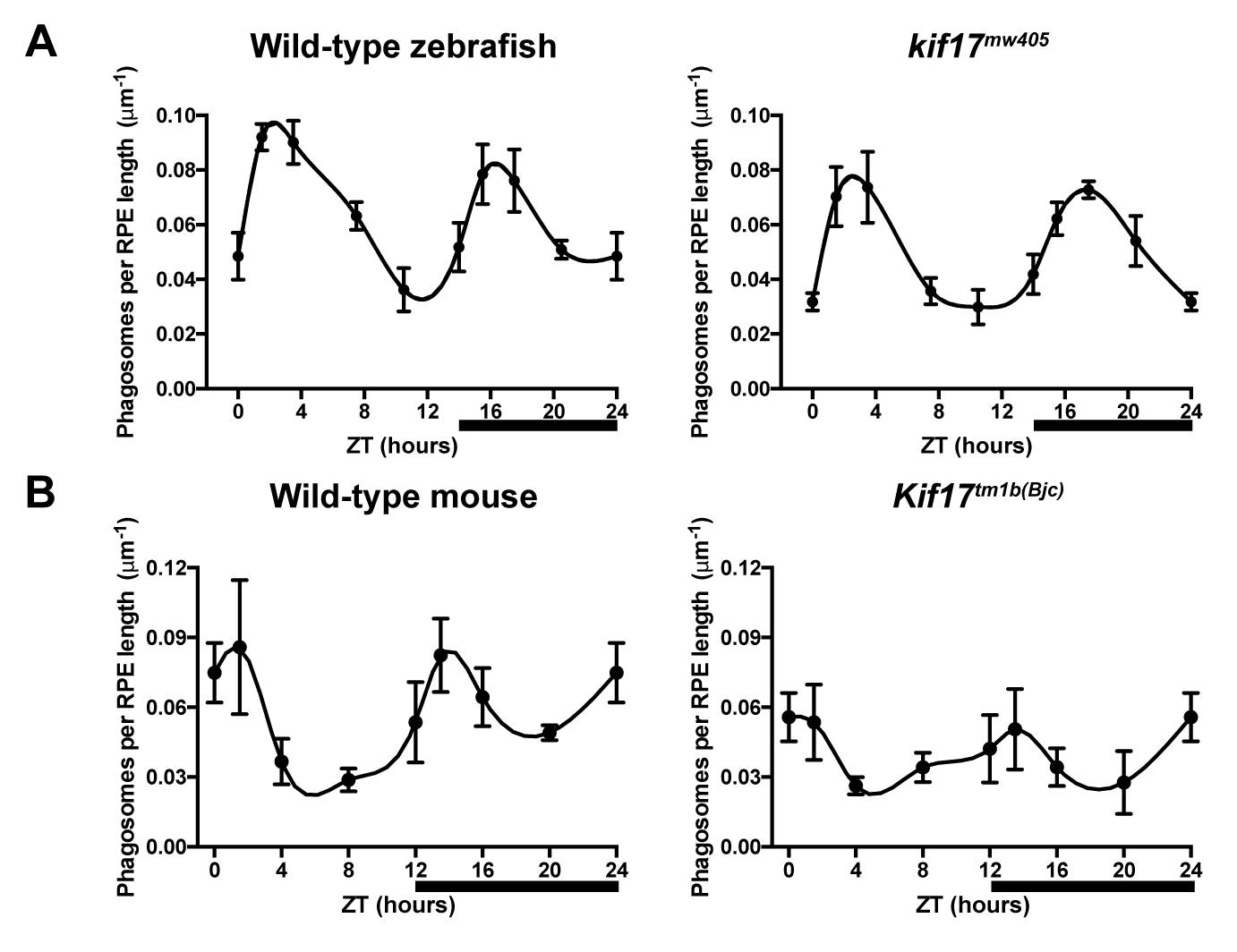


**Figure S9. Spline interpolation of zebrafish disc shedding data**

**(A)** For zebrafish, cubic spline interpolation with 40 segments was performed in Prism 6 (GraphPad) for both wild-type (left) and *kif17^mw405^* larvae (right). The spline fit (smooth curve) is plotted between the individual data points **(Figure 4A)** with the ZT 0 data-point repeated at ZT 24 for continuity. The 40 segments that make up the spline interpolation are depicted in **Table S1**. (**B)** For mice, cubic spline interpolation with 36 segments was performed in Prism 6 (GraphPad) for both wild-type (left) and *Kif17^tm1b(Bjc)^* mice (right). The spline fit (smooth curve) is plotted between the individual data points **(Figure 4D)** with the ZT 0 data-point repeated at ZT 24 for continuity. The 36 segments that make up the spline interpolation are depicted in **Table S2**.

**Table S1. Spline interpolation of zebrafish disc shedding**

| **ZT (hour)** | **Wild-type**  **Phagosomes**  **per RPE length (μm^-1^)** | ***kif17^mw405^***  **Phagosomes**  **per RPE length (μm^-1^)** |
| --- | --- | --- |
| **0.000000** | 0.04846007 | 0.03176749 |
| **0.61538464** | 0.06970063 | 0.050059214 |
| **1.2307693** | 0.086843595 | 0.065331385 |
| **1.846154** | 0.09601226 | 0.07470784 |
| **2.4615386** | 0.09718069 | 0.07781205 |
| **3.0769231** | 0.09359384 | 0.0763907 |
| **3.692308** | 0.088582076 | 0.072251156 |
| **4.3076925** | 0.08417915 | 0.066727296 |
| **4.923077** | 0.0802937 | 0.060376868 |
| **5.5384617** | 0.07663738 | 0.053685714 |
| **6.1538463** | 0.07292186 | 0.047139652 |
| **6.769231** | 0.06885877 | 0.041224513 |
| **7.384616** | 0.064159796 | 0.036426123 |
| **8.000000** | 0.058610607 | 0.033152018 |
| **8.615385** | 0.052522626 | 0.031253792 |
| **9.230769** | 0.04643475 | 0.030342463 |
| **9.846154** | 0.040886804 | 0.03002809 |
| **10.461539** | 0.036418606 | 0.029920727 |
| **11.076923** | 0.033538982 | 0.029756226 |
| **11.692308** | 0.032607157 | 0.029877627 |
| **12.307693** | 0.03393727 | 0.030810941 |
| **12.923078** | 0.037843477 | 0.033082213 |
| **13.538462** | 0.044639897 | 0.037217487 |
| **14.153847** | 0.054629073 | 0.043735616 |
| **14.769232** | 0.06670934 | 0.052285455 |
| **15.384616** | 0.07705194 | 0.060826212 |
| **16.000000** | 0.082046874 | 0.067334786 |
| **16.615385** | 0.081862435 | 0.07129211 |
| **17.230770** | 0.078301825 | 0.07282994 |
| **17.846155** | 0.07313512 | 0.07209517 |
| **18.461538** | 0.06744059 | 0.0694532 |
| **19.076923** | 0.061708957 | 0.06545508 |
| **19.692308** | 0.056412242 | 0.060657717 |
| **20.307693** | 0.052022483 | 0.05561803 |
| **20.923079** | 0.048971616 | 0.050858125 |
| **21.538462** | 0.047258947 | 0.04652451 |
| **22.153847** | 0.04661986 | 0.04253455 |
| **22.769232** | 0.046785947 | 0.03880232 |
| **23.384617** | 0.047488824 | 0.03524192 |
| **24.000000** | 0.048460085 | 0.031767424 |

**Table S2. Spline interpolation of mouse disc shedding**

| **ZT (hour)** | **Wild-type**  **Phagosomes**  **per RPE length (μm^-1^)** | ***Kif17^tm1b(Bjc)^* Phagosomes**  **per RPE length (μm^-1^)** |
| --- | --- | --- |
| **0.000000** | 0.07483927 | 0.0557309 |
| **0.6857143** | 0.08322848 | 0.05608264 |
| **1.371429** | 0.08632084 | 0.05430501 |
| **2.057143** | 0.07973781 | 0.04867601 |
| **2.742857** | 0.06562177 | 0.04036643 |
| **3.428572** | 0.04893692 | 0.03179881 |
| **4.114286** | 0.0346538 | 0.02539809 |
| **4.800000** | 0.02607785 | 0.02269443 |
| **5.485714** | 0.02243272 | 0.02302467 |
| **6.171429** | 0.02232806 | 0.02539572 |
| **6.857143** | 0.02437354 | 0.0288145 |
| **7.542857** | 0.02717883 | 0.03228791 |
| **8.228572** | 0.02936956 | 0.03483304 |
| **8.914286** | 0.03052984 | 0.03607716 |
| **9.600000** | 0.03172937 | 0.03659331 |
| **10.285710** | 0.03416565 | 0.03703589 |
| **10.971430** | 0.03903621 | 0.03805931 |
| **11.657140** | 0.04753855 | 0.04031798 |
| **12.342860** | 0.06068758 | 0.04438192 |
| **13.028570** | 0.07529862 | 0.04888019 |
| **13.714290** | 0.08404499 | 0.05052789 |
| **14.400000** | 0.08344802 | 0.04784337 |
| **15.085710** | 0.076635 | 0.04243867 |
| **15.771430** | 0.06734555 | 0.03621263 |
| **16.457140** | 0.05912336 | 0.03096518 |
| **17.142860** | 0.05323481 | 0.02734647 |
| **17.828570** | 0.04947703 | 0.02526495 |
| **18.514290** | 0.04762268 | 0.02461666 |
| **19.200000** | 0.04744443 | 0.02529766 |
| **19.885720** | 0.04871492 | 0.02720403 |
| **20.571430** | 0.05120917 | 0.03022358 |
| **21.257140** | 0.05471788 | 0.03418942 |
| **21.942860** | 0.05903814 | 0.03891236 |
| **22.628570** | 0.06396706 | 0.04420311 |
| **23.314290** | 0.06930176 | 0.04987241 |
| **24.000000** | 0.07483935 | 0.05573098 |
